# Comparative transcriptomic profiling reveals differentially expressed genes and important related metabolic pathways in shoots and roots of a Saudi wheat cultivar (Najran) under salinity stress

**DOI:** 10.3389/fpls.2023.1225541

**Published:** 2023-07-28

**Authors:** Norah Alyahya, Tahar Taybi

**Affiliations:** ^1^School of Natural and Environmental Sciences, Newcastle University, Newcastle upon Tyne, United Kingdom; ^2^Department of Biology, Faculty of Science, King Khalid University, Abha, Saudi Arabia

**Keywords:** salt stress, salt-tolerance, wheat, RNA-seq, differentially expressed genes (DEGs), salt enrichment pathways, Glutathione and phenylpropanoids

## Abstract

High salinity of soil is a threatening constraint for agricultural output worldwide. The adverse effects of salt stress on plants can be revealed in different manners, from phenotypic to genetic changes. A comparative RNA-Sequencing analysis was done in roots and shoots of bread wheat, Najran cultivar between plants grown under unstressed control condition (0 mM NaCl) and salt treatment (200 mM NaCl). More than 135 million and 137 million pair-end reads were obtained from root and shoot samples, respectively. Of which, the mapped reads to *Triticum aestivum* genome IWGSC_V51 ranged from 83.9% to 85% in the root and 71.6% to 79% in the shoot. Interestingly, a comparison of transcriptomic profiling identified that total number of significantly differentially expressed genes (DEGs) examined in the roots was much higher than that found in the shoots under NaCl treatment, 5829 genes were differentially expressed in the roots whereas 3495 genes in the shoots. The salt-induced change in the transcriptome was confirmed by RT-qPCR using a set of randomly selected genes. KEGG enrichment analysis classified all DEGs in both roots and shoots into 25 enriched KEGG pathways from three main KEGG classes: Metabolism, organismal systems and genetic information processing. According to that, the most significantly regulated pathways in the root and shoot tissues were glutathione metabolism and biosynthesis of secondary metabolites such as phenylpropanoids and galactose metabolism suggesting that these pathways might participate in wheat salt tolerance. The findings highlight the importance of the control of oxidative stress via Glutathione and phenylpropanoids and the regulation of galactose metabolism in the roots and shoots for salt-tolerance in wheat. They open promising prospects for engineering salt-tolerance in this important crop via targeted improvement of the regulation of key genes in the production of these compounds.

## 1 Introduction

Environmental stresses including water-stress and salt-stress represent a serious challenge to plant growth and crop productivity. It has been estimated that salt stress could severely restrict the productivity of about 30% arable land by 2050 (Wang et al., 2018). Ranked as the third most important crop, wheat (*Triticum aestivum*) is the staple food in many parts of the world. Unfortunately wheat production, is currently challenged by salinity stress that causes up to 40% yield loss, seriously compromising global food security (Singh et al., 2020).

A considerable number of studies has examined the harmful effects of salt stress on plant life (Borrelli et al., 2018; Hniličková et al., 2019; Tanveer et al., 2020). These harmful effects result first in growth inhibition, then accelerated development, senescence, and ultimately plant death. Accumulation of salts in soil causes a reduction in soil water potential leading to a hyperosmotic and hyper-ionic environment. High levels of salt cause a reduction in the amount of water and essential minerals that plant roots can take up from the soil (Seleiman et al., 2021). Consequently, reduced uptake of mineral nutrients results ultimately in nutritional deficiencies which eventually affect plant growth and reduce crop yield (Hajihashemi et al., 2009). Accumulation of toxic salts in the cell causes inhibition of enzymes which together with stomatal closure result in reduced photosynthetic efficiency, reduced cell elongation and division and thereby decreased biomass accumulation. Stomatal closure under stress results in the generation of reactive oxygen species (ROS), which are very harmful to cells at high concentrations. ROS toxicity causes cellular damage in the form of DNA mutations, protein degradation or lipid peroxidation (Apel & Hirt, 2004; Ahmad et al., 2010). Plants respond to oxidative stress, by synthesizing a variety of protective enzymes that act as an antioxidant scavenging systems (Ayvaz et al., 2016). Extensive research has indicated remarkable increases in the activity levels of scavenging enzymes, such as ascorbate peroxidase, guaiacol peroxidase, glutathione reductase, superoxide dismutase and catalase, in different wheat cultivars to mitigate against ROS-induced oxidative stress (Mandhania et al., 2006; Esfandiari et al., 2007; Rao et al., 2013). Other plant’s responses to salt-stress include osmotic adjustment, increased biosynthesis of secondary metabolites, which help plants stay hydrated and maintain ion homeostasis under stress conditions (Ashraf et al., 2008). All the above responses implicate thousands of genes which may be directly or indirectly involved in plant salt tolerance by regulating ion influx and efflux (Li et al., 2020), production and accumulation of osmotica or compatible solutes (Singh et al., 2018), biosynthesis of signaling and regulatory enzymatic and nonenzymatic elements (Thabet et al., 2021). To understand the complexity of the various plant’s responses to salt stress, gene expression profiling can be used to identify salt-responsive genes. Many studies used transcriptomics to unravel the genes and associated metabolic pathways and biological functions involved in tolerance of environmental stress in wheat (Goyal et al., 2016; Amirbakhtiar et al., 2019; Luo et al., 2019). Despite the latest interest in transcriptome analysis in wheat under salt stress, all available studies have focused either on roots or shoots. No research has analyzed the effect of salt-stress on gene expression in both roots and shoots simultaneously. In this study, a global RNA-Seq analysis was done in the roots and shoots of a salt-tolerant wheat cultivar (Najran) under two conditions: control (0 mM NaCl) and salt treatment (200 mM NaCl). The current study identified differentially expressed genes revealing key biological pathways and processes involved in wheat responses to salt stress in root and shoot tissues, providing important insights into the molecular mechanisms underlying salt tolerance in this species. To our knowledge this is the first work where salt-induced changes in the transcriptome were analyzed in roots and shoots of the same wheat plants at the same time providing an integrated view of the response of wheat plants to salt-stress. The findings show that the control of oxidative stress via the production of antioxidants including glutathione and phenylpropanoids and galactose metabolism in roots and shoots are key pathways in the salt-tolerance trait in wheat. They offer a promising avenue for developing salt-tolerance in wheat via marker-assisted breeding or via genetic engineering technologies including gene editing to optimize the expression of targeted genes in these pathways.

## 2 Material and methods

### 2.1 Plant growth and salt stress treatment

Seeds of Najran wheat cultivar (accession No. 193) were obtained from Ministry of Environment, Water & Agriculture, Saudi Arabia. Cold-stratified seeds were germinated as three seeds per pot in 2L pots filled with 2:1:1 (v/v/v) mix of John Innes soil compost No. 2, vermiculite, and grit sand, respectively. After germination plants were divided into two groups, each group having a salt-treated batch and water-control batch.

The first group consisted of (1) unstressed control plants, watered with tap water and (2) salt-treated plants, watered with 200 mM NaCl solution. These plants were grown for 4 weeks from germination to full vegetative growth. Plants from this group were used to measure growth and extract RNA for sequencing.

The second group consisted of (1) unstressed control plants, watered with tap-water and (2) salt-treated plants watered with 100 mM NaCl solution. These plants were grown until flowering and seed production. Spikes were harvested when seeds became dry.

All plants were watered every other day and kept under controlled conditions; 16 h light at 20°C, 8 h dark at 15°C and 70% humidity.

### 2.2 Growth and yield analysis

Roots and shoots were harvested separately at midday, and roots washed thoroughly to remove soil and dried with paper towel. Different growth parameters including root and shoot fresh weight, dry weight and length were recorded. Roots and shoots were grouped into three replicates (each sample had a duplicate of plants), snap frozen in liquid nitrogen and stored at -80°C, they were later ground to a fine powder under liquid nitrogen to be used in RNA extraction. Dry weight was determined after drying plant tissues in an oven at 80°C for 7 days. To evaluate the impact of salt-stress on yield, the number of spikes and seeds was determined, and seed weight measured in control and salt-treated plants. Root and shoot Dry weight and number of spikes per plant and number of seeds per plant obtained.

### 2.3 RNA extraction and qualification

Total RNA was extracted from twelve root and shoot samples using Plant/Fungi Total RNA Purification Kit, according to the manufacturer’s instructions (Cat. 25800, Norgen, Canada) and treated with RNase-Free DNase I (Cat. 25710, Norgen) during RNA purification. RNA purity and quality were assessed using NanoDrop 1000 spectrophotometer whereas its integrity was examined by RNA 6000 Nano Kit for Agilent 2100 Bioanalyzer (Agilent Technologies, Waldbronn, Germany). High quality RNA samples with RIN ≥ 7.2 were sent to Admera Health LLC (New Jersey, USA) for cDNA library construction and sequencing.

### 2.4 Illumina library construction and transcriptome sequencing

According to Admera Health LLC, the quality of 20 µl RNA samples was assessed by High Sensitivity RNA Tapestation (Agilent Technologies Inc., California, USA) and quantified by Qubit 2.0 RNA HS assay (ThermoFisher, Massachusetts, USA). Paramagnetic beads coupled with oligo d(T)25 were combined with total RNA to isolate poly (A)+ transcripts based on NEBNext^®^ Poly(A) mRNA Magnetic Isolation Module manual (New England BioLabs Inc., Massachusetts, USA). Prior to first strand synthesis, samples were randomly primed (5´ d(N6) 3´ [N=A,C,G,T]) and fragmented based on manufacturer’s recommendations. The first strand was synthesized with the Protoscript II Reverse Transcriptase with a longer extension period, approximately 40 minutes at 42°C. All remaining steps for library construction were done according to the NEBNext^®^ Ultra™ II *Non-Directional* RNA Library Prep Kit for Illumina^®^ (New England BioLabs Inc., Massachusetts, USA). Final libraries quantity was assessed by Qubit 2.0 (ThermoFisher, Massachusetts, USA) and quality was assessed by TapeStation D1000 ScreenTape (Agilent Technologies Inc., California, USA). Final library size was about 430bp with an insert size of about 300bp. Illumina^®^ 8-nt dual-indices were used. Equimolar pooling of libraries was performed based on QC values and sequenced on an Illumina^®^ Novaseq S4 (Illumina, California, USA) with a read length configuration of 150 PE for *40*M total reads per sample (*20*M in each direction).

### 2.5 RNA-seq data analysis

The workflow of RNA-Seq data analysis started with the pre-processing of the raw reads and ended with identifying enriched KEGG pathways for the DEGs (**Figure S1**). All data presented and discussed in this study have been deposited in NCBI’s Gene Expression Omnibus and are accessible through GEO Series accession number GSE225565 (https://www.ncbi.nlm.nih.gov/geo/query/acc.cgi?acc=GSE225565).

#### 2.5.1 Quality control and read quantification

Raw sequences from FASTQ files generated by Illumina were assessed with FastQC (version 11.8, http://www.bioinformatics.babraham.ac.uk/projects/fastqc). A total of 136281942 and 138608660 paired-end reads were obtained from twelve control and salt stressed samples of root and shoot tissues, respectively (**Table 1**). Trimmomatic (version 0.36, http://www.usadellab.org/cms/?page=trimmomatic) was used for trimming out contaminating adaptor sequences and producing filtered reads (Bolger et al., 2014).

**Table 1:** Summary of sequencing output, clean reads and mapping on the genome. R indicates root and S indicates shoot.

Sample	Total reads	High quality reads	Mapped reads
Ctrl_R_1	23001104	22838034	19425012
Ctrl_R_2	26022495	25836862	21983090
Ctrl_R_3	21950892	21726586	18437481
Salt_R_1	24008611	23818150	20066908
Salt_R_2	20462131	20265845	17006769
Salt_R_3	20836709	20702466	17395220
Ctrl_S_1	20340235	20188148	14449739
Ctrl_S_2	27874023	27720205	21256010
Ctrl_S_3	26033823	25804393	19259820
Salt_S_1	20727638	20590369	15766118
Salt_S_2	20869668	20721980	16374493
Salt_S_3	22763273	22575139	17764005

High-quality reads were quantified against transcripts derived from the Ensembl *T. aestivum* genome (version 51, https://plants.ensembl.org/Triticum_aestivum/Info/Index). Salmon (version 0.12.0, https://combine-lab.github.io/salmon); a tool which performs ‘quasi-alignment’ was used to quantify expression of transcripts from RNA-Seq data via read abundances. To quantify the levels of gene expression (gene-level counts), the R package ‘tximport’ (version 1.18.0, http://bioconductor.org/packages/release/bioc/html/tximport.html) was used.

#### 2.5.2 Differential gene expression analysis

Differentially expressed genes (DEGs) were identified using the R package DESeq2 (version 1.30.1, http://bioconductor.org/packages/devel/bioc/vignettes/DESeq2/inst/doc/DESeq2.html). DESeq2 estimates fold change between experimental conditions (**Table 2**) in the root and shoot samples using a Negative Binomial GLM (General Linear Model) with a logarithmic link function. According to that, genes which have base 2 logarithmic fold change ≥1 and a cut-off of adjusted P-value (false discovery rate (FDR)) < 0.01 were considered as upregulated genes, whereas genes with a fold-changes ≤ −2 (Log_2_ ≤ −1) have been indicated as downregulated genes.

**Table 2:** Number of total differentially expressed genes (DEGs) and up/down-regulated genes in the root and shoot tissues of Najran wheat cultivar under 0 mM NaCl (control) and 200 mM NaCl (salt treatment) conditions.

	Total DEGs	Up-regulated	Down-regulated	Up_differences % ratio	Down_differences % ratio
Salt treated Root vs Control Root	5829	2226	3603	38.19	61.81
Salt treated Shoot vs Control Shoot	3495	1473	2022	42.15	57.85

#### 2.5.3 GO terms analysis

The GO terms enrichment analysis was done by searching the Gene Ontology resource (http://geneontology.org/) using the obtained DEGs against the wheat repository. Testing for enriched GO terms was carried out using the hyper GTest function from the R package GO stats, with a p-value test at cut-off of 0.05.

#### 2.5.4 Functional enrichment analysis

The Kyoto Encyclopedia of Genes and Genomes (KEGG) was searched to identify enriched KEGG pathways for the DEGs. KEGG pathways for *T. aestivum* were obtained using the R package ‘KEGGREST’ (version 1.34.0, https://bioconductor.org/packages/release/bioc/html/KEGGREST.html). Protein sequences for wheat genes were downloaded in FASTA format using the ID mapping tool from UniProt (https://www.uniprot.org/uploadlists/). KEGG’s ‘GhostKOALA’ search (https://www.kegg.jp/ghostkoala) was used to find KEGG orthology IDs for wheat genes based on the UniProt FASTA download. The KEGG orthology numbers enabled a mapping from wheat genes to KEGG pathways. Enriched KEGG pathways for the lists of significantly DEGs were calculated using a hypergeometric test with the corrected P-value < 0.05.

### 2.6 Validation of RNA-sequencing results using quantitative real-time PCR analysis

To validate the gene expression profiles obtained from the analysis of RNA-Seq data, eight genes differentially expressed under salt-stress were randomly chosen and their transcript levels monitored by RT-qPCR using the same RNA samples to RNA-Seq. The genes included four genes upregulated in both roots and shoots, two genes down-regulated in roots and shoots, one gene upregulated in roots and downregulated in shoots and one gene down regulated in roots and up-regulated in shoots (**Table S1**). Gene-specific primers (**Table S2**) for RT–qPCR (18–21 bp) were designed using Primer3(v. 0.4.0). cDNA was synthesized from twelve purified RNA samples using Tetro™ cDNA Synthesis Kit (Bioline, UK) according to the manufacturer’s procedure using Oligo dT primer. The SensiFAST™ SYBR Hi-ROX Kit (Bioline, UK) was used to perform qPCR in Rotor Gene Q5 & Qiagility Robot instrument (Qiagen, UK) based on the manual of SYBR Hi-ROX Kit (Bioline, UK) using cDNA as template. Normalization of gene expression level was done using CJ705892 gene as a housekeeping (internal control) gene from wheat (Dudziak et al., 2020). Primer efficiency was determined using serial dilutions of the cDNA for each gene, it ranged from 95% to 103%. Relative gene transcript levels of the target genes were quantified using the standard 2−^(δδCt)^ method (Livak & Schmittgen, 2001).

## 3 Results

### 3.1 Plant growth and yield

**Figure 1:**
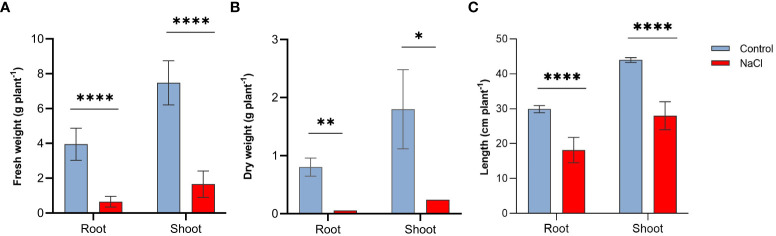
Effect of salt-stress on **(A)** fresh weight, **(B)** dry weight, and **(C)** lengths of the root and shoot of wheat *(Triticum aestivum)* (n=6 +/- S.E) plants subjected to salt-stress (200mM NaCl) or watered with tap water only (control). Columns with asterisks refer to the significant difference between control and salt stressed samples assigned using Independent T-Test (*P* ≤ 0.05). ns means P-value > 0.05, * means P ≤ 0.05, ** means P ≤ 0.01, **** means P ≤ 0.0001.

Salt stress resulted in important reductions in all growth parameters tested in this study. Plants subjected to salt-stress (200 mM NaCl) have shown 8.3-, 7.8-fold decrease in their root and shoot fresh weight, respectively (**Figure 1A**). Similarly, salt-stress caused a 9.3- and 8.7-fold decrease in the root and shoot dry weight, respectively in these plants (**Figure 1B**). Salt stress also affected plant’s height, as shown in **Figure 1C** salt-stressed plants have shown approximately 39% and 36% decline in the length of roots and shoots respectively.

**Figure 2:**
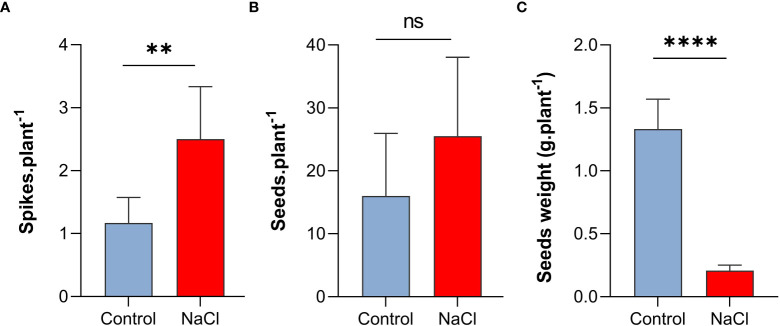
Effect of salt-stress on **(A)** spikes number, **(B)** seeds number, and **(C)** seeds weight of wheat *(Triticum aestivum)* (n=6 +/- S.E). plants subjected to salt-stress (200mM NaCl) or watered with tap water only (control). Columns with asterisks refer to the significant difference between control and salt stressed samples assigned using Independent T-Test (*P* ≤ 0.05). ns means P-value > 0.05, * means P ≤ 0.05, ** means P ≤ 0.01, **** means P ≤ 0.0001.

Interestingly, despite the negative effect on plant growth, salt-stress with 100 mM NaCl resulted in increased number of spikes and seeds per plant. As shown in **Figure 2A**, spike number per plant increased to more than two-folds under salt-stress compared to control plants (P < 0.01). Similarly, seed number per plant increased under salt-stress by 62.5% (**Figure 2B**). In contrast, the weight of seeds (0.21 g) decreased by 84% of the control (1.33 g) (P < 0.001) under saline conditions (**Figure 2C**)

### 3.2 Transcriptome profiling and sequencing statistics

In order to investigate the regulatory mechanism of wheat responses to salinity stress at transcriptional level, RNA sequencing was carried out in the root and shoot tissues of Najran cultivar. Three biological replicates for each tissue and treatment were used in Illumina high-throughput sequencing. The number of raw reads obtained varied from 20.34 million to 27.87 million for 12 samples with a mean of 22.90 million. A total of 272788177 high-quality pair-end reads (99% of the raw reads) were generated from all samples; 135187943 and 137600234 reads from roots and shoots (**Table 1**), respectively. All the clean reads were quantified against transcripts of *T. aestivum* genome IWGSC_V51 (accessed Nov. 2021), resulting in mapped reads ranging from 83.9% to 85% (17006769 to 21983090 reads) in the root and 71.6% to 79% (14449739 to 16374493 reads) in the shoot (**Table 1**).

### 3.3 Differential gene expression analysis

**Figure 3:**
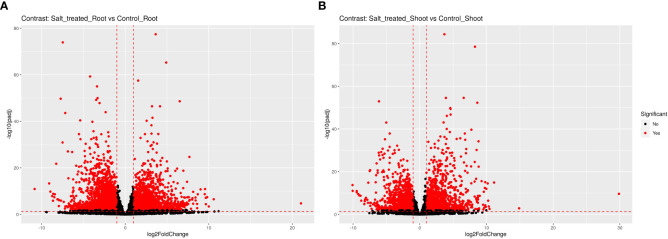
The volcano plot (log_2_ FC ≥1 or ≤ -1, p-adj ≤ 0.01) of differentially expressed genes in roots and shoots of Wheat (Najran) under salt-stress (200mM NaCl). DEGs in **(A)** salt-treated root versus control root, **(B)** salt-treated shoot versus control shoot. Red dotes towards the right indicate statistically significant-upregulated genes, red dotes towards the left indicate statistically significant-downregulated genes and black dotes indicate non-significant genes.

Transcript abundance of each gene expressed under 0 or 200 mM NaCl conditions and mapped was normalized, then the significance of difference in transcript abundance in the root and shoot tissues was determined based on the thresholds of adjusted P-value (FDR < 0.01). Interestingly, a total of 5829 genes were differentially expressed in the roots under salt treatment, including 38.19% up-regulated genes and 61.81% down-regulated genes (as obviously depicted in the volcano plot **Figure 3A**). On the other hand, 3495 DEGs were revealed between the control and salt treated shoots; 42.15% of them were up-regulated, while 57.85% were down-regulated (**Figure 3B**). Only 1205 from 5829 genes expressed in roots and 733 from 3495 genes in shoots had annotated functions (**Table 2**).

**Figure 4:**
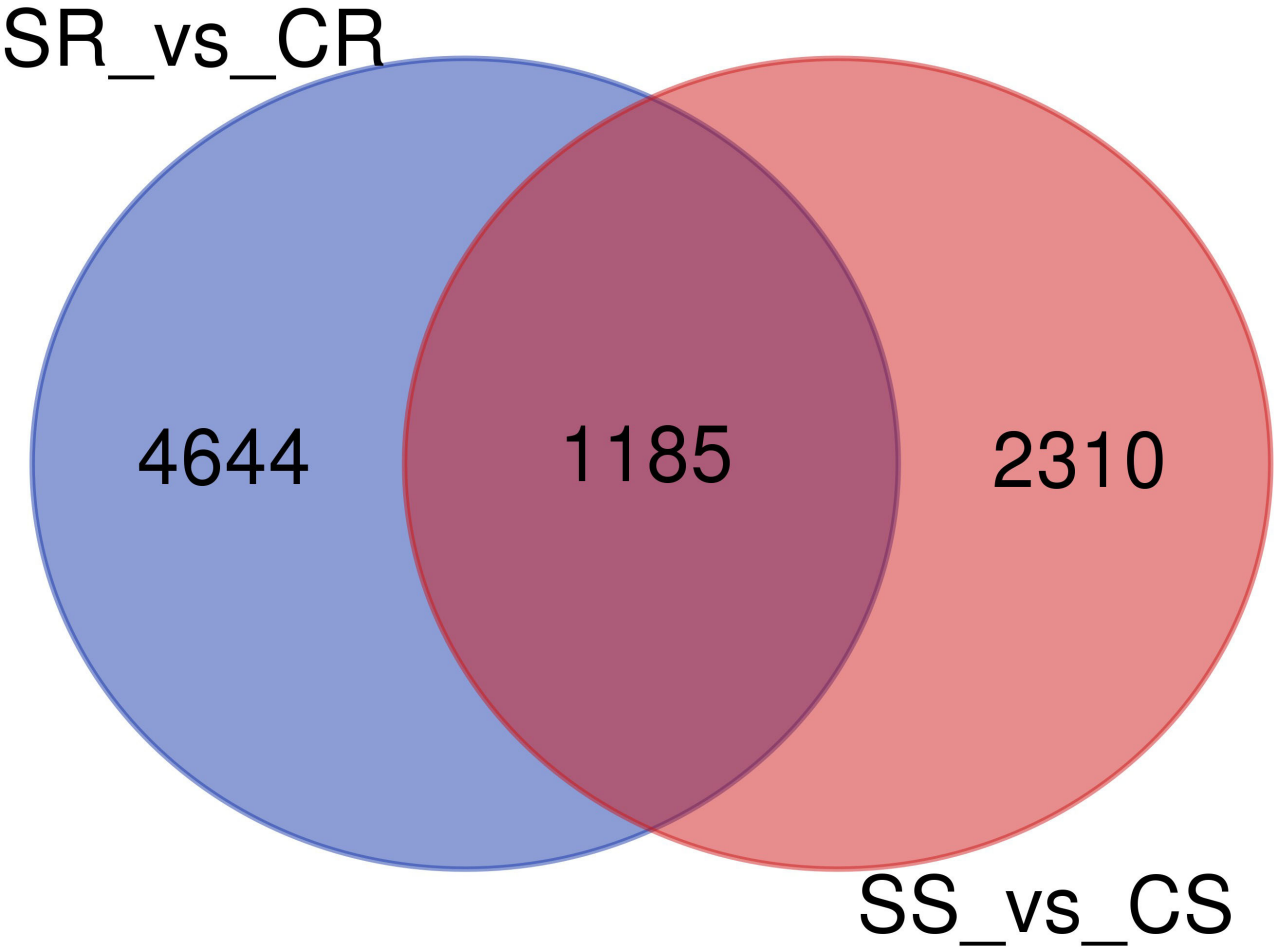
Venn diagram of differentially expressed genes (DEGs)in roots and shoots of Najran wheat *(Triticum aestivum)* under salt-stress (200mM NaCl). The numbers in the Venn diagram indicate DEGs in salt treated-roots vs. control-roots (SR_vs_CR), root and salt-treated shoots vs. control-shoots; (SS_vs_CS).

The number of genes that were identified as DEGs in roots and shoots was compared and the overlap of DEGs in the two organs analyzed using a Venn diagram (**Figure 4**). The Venn diagram showed that only 1158 genes were overlapping genes expressed in roots and shoots under salt-stress conditions.

### 3.4 Cluster analysis of RNA-sequencing data

**Figure 5:**
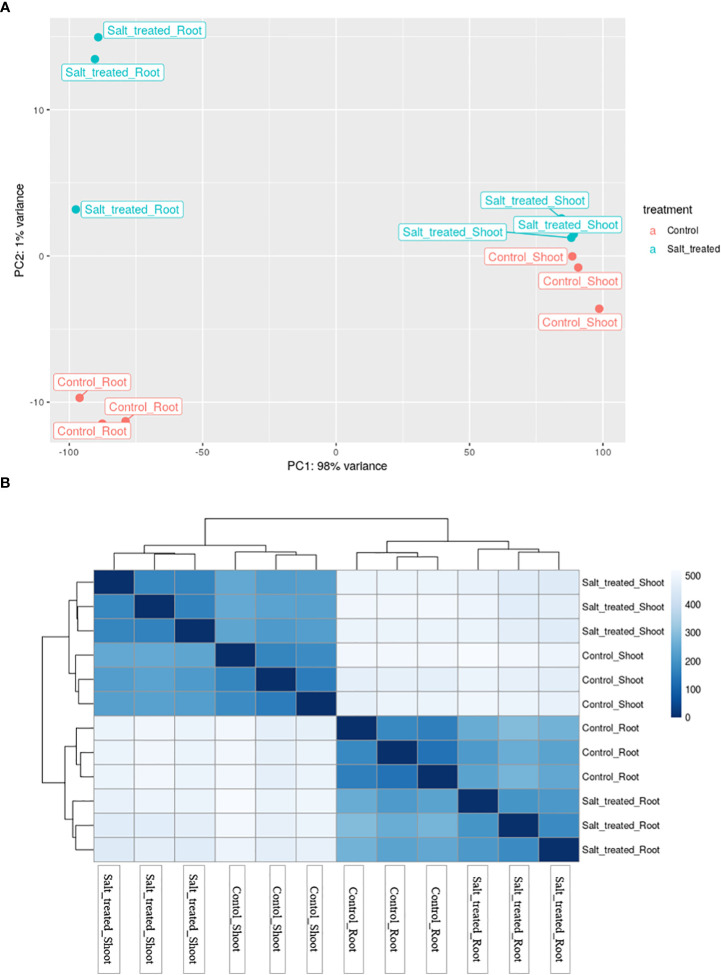
**(A)** Principal component analysis (PCA) of differential gene expression in Najran wheat (*Triticum aestivum*) subjected to salt-stress (200mM NaCl). Samples showing the relationship between salt stressed and control roots and shoots. Blue color refers to control samples and orange color to salt treated samples. **(B)** Heatmap showing the clustering and relationship of gene expression in salt-stressed and control roots and shoots, each sample being represented by three replicates. Darker color refers to more similarity between samples.

To have an overview of the relationship of expression patterns between the control and salt stressed samples as well as between the roots and shoots, Principal Component Analysis (PCA) and hierarchical clustering methods were used. PCA, showed that 98% variance was observed between the root and shoot samples (**Figure 5A**). Moreover, these two distinct groups formed based on their expression profile; one group comprised control and salt-treated roots and the other group comprised shoots from non-stressed and stressed plants. On the other hand, the PCA plot in **Figure 5A** demonstrated that the outlier (Salt-treated root) at the middle west of the plot is almost entirely responsible for the variation seen in the second principal component (1% of the variation in the entire dataset).

The heatmap visualization of gene expression, combined with clustering method grouped the 12 samples based on similarity of their gene expression pattern and these classifications were consistent with the PCA findings. The color and intensity of heat map boxes was used to represent the similarity in gene expression, the darker the blue color, the more similarity between samples. The heatmap analysis indicated the notable difference between the control and salt treated samples in both roots and shoots and a massive difference between the gene expression profiles of the roots and shoots (**Figure 5B**). These variations in gene expression patterns among wheat samples are a result of exposing plants to salt stress as well as differences in plant organs.

### 3.5 GO terms enrichment of DEGs

To determine the functional meaning of the salt-induced changes in the transcriptome in roots and shoots of Najran Wheat, we performed the GO terms enrichment for the DEGs in the two organs.

**Figure 6:**
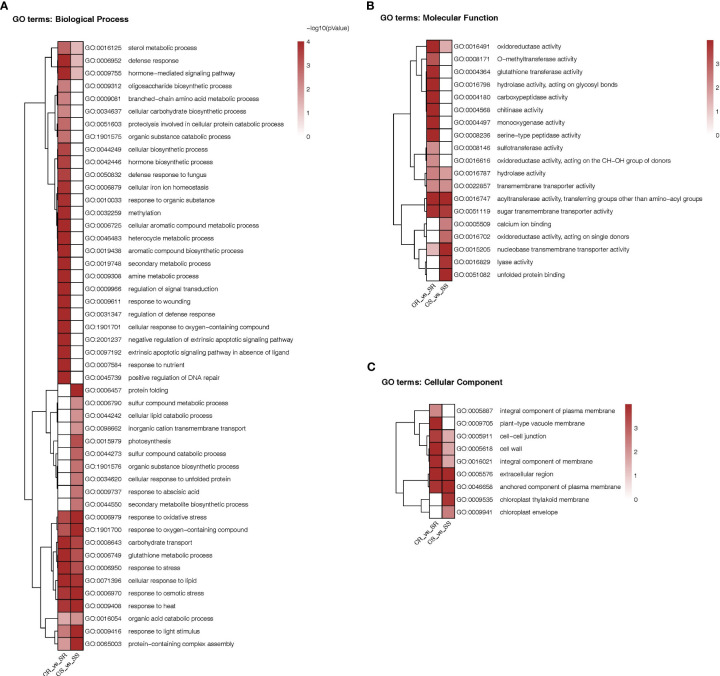
Heatmap comparison of Gene Ontology classifications of DEGs in roots and shoots of Najran Wheat plants under salt stress (200 mM NaCl) versus unstressed control. The heatmaps representing GO terms based on **(A)** biological process (45 categories), **(B)** molecular function (19 categories) and **(C)** cellular component (9 categories). The color scale indicates different levels of significance of enriched terms (P-value < 0.05).

As shown in the heatmap of **Figure 6**, the DEGs in roots and/or shoots were associated with 76 functional categories in total. The DEGs were associated with 48 “biological process” categories, 19 “molecular function” categories and 9 “cellular component” categories. Exactly 35 categories were present only in roots and 16 were present only in shoots while 25 categories were present in both organs. Among the categories enriched in roots there were processes involved in cell ionic homeostasis, oxidative stress responses including Glutathione synthesis, osmotic stress response, hormonal signaling, carbohydrate transport etc. Among the categories enriched in shoots there were protein folding, photosynthesis, synthesis of secondary metabolites, response to Abscisic acid, hormonal-signaling. Among the categories enriched in both organs we find response to oxidative stress response, glutathione synthesis and carbohydrate transport.

### 3.6 KEGG enrichment analysis of DEGs and functional annotations

**Figure 7:**
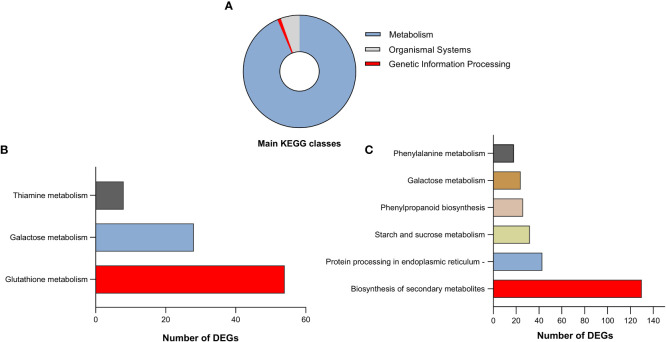
**(A)** Categorization of the identified differentially expressed genes in both roots and shoots of Najran wheat (*Triticum aestivum*) in three chief KEGG classes. The top significantly enriched pathways of identified DEGs in roots **(B)** and shoots **(C)** in response to salinity stress.

To identify which biological pathways for the DEGs were potentially enriched in the roots and shoots of Najran cultivar transcriptome, KEGG enrichment analysis was done for all pairwise comparisons. The KEGG analysis revealed that all DEGs in both roots and shoots could be classified into 25 enriched KEGG pathways which located in three main KEGG classes; metabolism, organismal systems; environmental adaptation and Genetic information processing; folding, sorting and degradation (**Figure 7A**, **Tables S3, S4**). Among them, the number of DEGs was the largest in metabolism category, which included 4044 genes involved in different pathways such as biosynthesis of secondary metabolites, Amino acid metabolism, Carbohydrate metabolism, Metabolism of cofactors and vitamins and other compounds.

Pathways relating to glutathione metabolism, galactose metabolism and thiamine metabolism were significantly upregulated in the roots under salt stress (**Figure 7B**), where glutathione metabolism had the highest number of DEGs (**Figure S2**). In contrast, the significantly enriched pathways in the salt treated shoot were biosynthesis of secondary metabolites, protein processing in endoplasmic reticulum, starch and sucrose metabolism, phenylpropanoid biosynthesis, galactose metabolism and phenylalanine metabolism pathways (**Figure 7C**). Most of DEGs were related to biosynthesis of secondary metabolites such as genes associated with phenylpropanoids (**Figure S3**).

Comparing salt-stress to control plants revealed three and six different pathways preferably enriched in salt treated roots vs control roots and salt treated shoots vs control shoots, respectively (**Table S4**). Interestingly, the only common pathway between salt treated roots vs control roots and salt treated shoots vs control shoots was galactose metabolism (**Figure S4**), which is part of carbohydrate metabolism and has been reported to have a role in salt tolerance (Darko et al., 2019).

### 3.7 Validation of RNA-seq results using quantitative real-time PCR analysis

**Figure 8:**
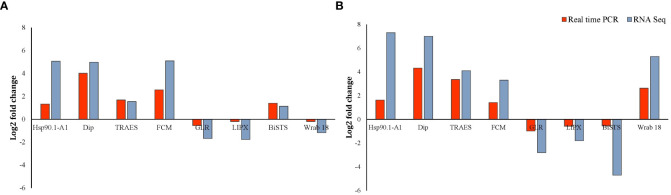
RT-qPCR validation of the salt-induced transcript change in Najran wheat (*Triticum aestivum*) subjected to salt- stress (200 mM NaCl). Log2 fold change in transcript levels is shown in root **(A)** and shoot **(B)** for eight genes including four up-regulated genes in roots and shoots (TaHSP 90, TaDip, Tres CFD, TaCFM), two genes down regulated in roots and shoots (TaGLR and Ta LIPX), one gene upregulated in roots and down regulated in shoots (taBiSTS) and one gene down regulated in roots and up-regulated in shoots (TaWrab18). Red bars represent qPCR results, whereas blue bars represent the results of RNA sequencing.

To validate the change in transcript-levels revealed by RNA-Seq data, transcript levels of eight DEGs in both roots and shoots of Najran wheat were measured by RT-qPCR. The results of this analysis (**Figure 8**) confirmed that change (up or down-regulation depending on gene) in transcript levels was consistent with that obtained by RNA-Seq for the selected genes including heat shock protein 90, dirigent protein, delta-1-pyrroline-5-carboxylate synthase, flavin-containing monooxygenase, glutamate receptor, lipoxygenase, bidirectional sugar transporter SWEET and ABA inducible protein. The log2 fold change in transcript levels for the eight randomly selected genes measured by RT-qPCR and RNA-Seq is shown in **Figure 8**.

## 4 Discussion

### 4.1 Growth and yield

Salt-stress caused a significant reduction in root and shoot growth of Najran wheat. This decrease in growth and biomass is common to most non-halophytic plants including wheat which is considered as a salt-tolerant non halophyte. A previous study compared the effect of salt-stress on the growth of three wheat cultivars from Saudi Arabia including Najran, Qiadh, Mebiah, the results concluded that among the three cultivars, Najran was the least impacted by salinity (Alyahya and Taybi unpublished). Similarly, Alfagham (2021) compared the effect of water-stress on the growth of six wheat cultivars including Najran, Jaizan, Sama, Rafha, Ma’ayah and Najd and concluded that Najran was the least impacted by water-stress. In contrast to growth and biomass, salinity has increased the number of spikes and seeds per plant in Najran wheat. Although the seeds were smaller, they maintained a full-germination rate compared to seeds obtained from unstressed plants. This response clearly suggests a high degree of adaptability of this wheat cultivar to survive and maintain itself under stressful conditions. Dadshani et al. (2019) have shown that salt-stress decreases root and shoot growth (dry weight and length) in three wheat genotypes, SBLs, Zentos, Syn86. Moreover, high soil concentrations of some mineral nutrients like Boron, impact negatively growth in different wheat cultivars (Pandey et al., 2022; Khan et al., 2023).

### 4.2 Differential gene expression

Global profiling of the transcriptome of roots and shoots of Najran wheat revealed profound changes in gene expression under salt-stress as shown by the high number of DEGs. The expression of about 6.5% and 3.9% of the estimated 90000 wheat genes changed in roots and shoots respectively under salt-stress. Among the identified DEGs, candidate genes related to key pathways with important roles in adaptation and responses to salt stress were identified. It is worth noting the levels of transcriptional changes under abiotic stresses vary between plant tissues, this being observed to a greater extent in tissues sensing stress early rather than those subsequently detecting it. Roots of drought-treated Arabidopsis plants, undergo higher levels of transcriptional alterations compared to shoots under water-stress, suggesting that the physiological adjustments induced in roots were deeper to those induced in shoots (Bashir et al., 2018). Goyal et al. (2016) have shown that 17,911 genes were differentially expressed under salt-stress in roots of Kharchia wheat. Similarly, Amirbakhtiar et al. (2019) have shown that 5128 were differentially expressed under salt-stress in roots of Arg wheat. Among these genes, there were genes for sensing and signaling of salt stress such as genes that encode calcium transporters (Ta.ANN4, Ta.ACA7, Ta.NCL2 and Ta.GLR) and SOS1 (Na^+^/H^+^ antiporter), genes coding for transcriptional regulators such as transcription factors (MYB, bHLH, AP2/ERF, WRKY, NAC, and bZIPs) as well as genes related to salt stress adaptation, including genes coding for LEA proteins, Aquaporins, P5CS, dehydrins, ABA and K+ transporters (Ta.ABAC15, Ta.HAK25), catalases, glutathione-S-transferases.

Roots as the first organ encountering salinity in the surrounding media often respond by deploying specific responses to maintain water and nutrient uptake while limiting the uptake of toxic ions and potentially extruding them (Rajaei et al., 2009). On contrast, plant shoots detect and respond to salt-stress at a later stage, they might deploy specific responses to keep cells hydrated and protect metabolism from inhibition including photosynthetic reactions to keep growth. Shoots have higher sensitivity to salinity than the roots (Esechie et al., 2002).

### 4.3 Functional enrichments of the DEGS

Functional enrichment of the DEGs in roots and shoots of Najran wheat revealed important biological and molecular functions that may be key to the survival and development of this wheat cultivar under salinity. Genes associated with glutathione metabolism, galactose metabolism and thiamine metabolism were differentially regulated in roots of salt treated Najran wheat compared to the control. The highest number of DEGs was in glutathione metabolism pathway. These DEGS included fifty genes coding for glutathione S-transferase (GST) and one gene coding for glutathione dehydrogenase. Three genes encoded glutathione synthase (GSS) were however downregulated in response to salinity stress. Exposure of plants to salt stress may increase the production of reactive oxygen species (ROS) which might lead to cellular damage via protein denaturation, peroxidation of membrane lipids, DNA mutation, pigment breakdown and carbohydrate oxidation (Noctor and Foyer, 1998; Ahmad et al., 2010). To alleviate ROS-destructive effects, plants have evolved ROS-scavenging systems, including regulatory enzymes eg. glutathione transferase (GT), ascorbate peroxidase (APX), catalase (CAT), and nonenzymatic elements (metabolites such as phenolics, glutathione, flavonoids, ascorbic acid, carotenoid). The detoxifying role of these enzymes and antioxidants has been reported to enhance salt tolerance in many plants such as GST in Arabidopsis (Qi et al., 2010), glutathione peroxidase (GPX) in rice (Paiva et al., 2019), phenolic acids in cabbage (Linić et al., 2019), APX, CAT, superoxide dismutase (SOD) and peroxidase (POD) in Tobacco (Li C et al., 2020) and flavonoids in *Ginkgo biloba* (Xu et al., 2020). The significantly enriched pathways in shoots of salt-treated plants included biosynthesis of secondary metabolites, protein processing in endoplasmic reticulum, starch and sucrose metabolism, phenylpropanoid biosynthesis, galactose metabolism and phenylalanine metabolism pathways. Most DEGs in shoots of Najran wheat were related to biosynthesis of secondary metabolites pathway. Secondary metabolites play important roles in plants acclimation to different environmental stresses. In this study, genes associated with phenylpropanoid, galactose metabolism, fatty acid elongation, flavonoid biosynthesis, starch and sucrose metabolism and lignin synthesis pathways were shown to be significantly regulated in shoots under NaCl stress. As an example of the salt-regulated genes in phenylpropanoid biosynthesis there were genes encoding phenylalanine ammonia-lyase (PAL), two isogenes encoding for caffeoyl shikimate esterase (CSE), one isogene encoding for cinnamoyl-CoA reductase (CCR) and one isogene encoding ferulate-5-hydroxylase (CYP84A). All these genes have previously been documented to participate in salt tolerance in (Amirbakhtiar et al., 2021; Kong et al., 2021). In phenylpropanoid biosynthesis, PAL is involved in the first step of synthesizing trans-cinnamate from L-phenylalanine and acts as one of the antioxidative components produced under stress to minimize oxidative damage induced by salt stress (Gholizadeh and Kohnehrouz, 2010). The final products in this metabolic pathway are lignin components, including syringyl lignin, 5-hydroxy-guaiacyl-lignin and guaiacyl lignin. It is widely known that plants synthesize lignin to maintain the structural integrity of cell wall and the rigidity of the stem helping the plants to cope with various environmental stresses (Rao et al., 2017). More recent work highlighted the important regulatory role of NAC transcription factor (AgNAC1) in lignin biosynthesis which ultimately enhances salt tolerance in *Arabidopsis thaliana* plants (Duan et al., 2020). In the present study, one NAC domain-containing gene was significantly up-regulated in wheat shoots and five genes were down-regulated in the roots. Another study conducted on *Betula platyphylla* suggested the positive correlation between overexpression of BpNAC012 and lignin biosynthesis and its crucial role in the tolerance of both salt and osmotic stresses (Hu et al., 2019).

Luo et al. (2019) carried out functional enrichment analysis for DEGs induced under salinity stress in new leaf, old leaf, and root tissues of two wheat varieties, Zhongmai 175 and Xiaoyan 60, they found that metabolic pathways including those for phenylpropanoid biosynthesis, biosynthesis of secondary metabolites, benzoxazinoid biosynthesis and starch and sucrose metabolism were significantly enriched in the three organ types. DEGs under drought were classified into 5 key categories of KEGG pathways in roots (Amirbakhtiar et al., 2019) and leaves (Amirbakhtiar et al., 2021) of Arg wheat cultivar namely metabolism, genetic information processing, environmental information processing, cellular processes and organismal systems. Metabolism in these studies has been reported to have the greatest enrichment, far larger than the other enriched pathways. Similarly, our findings revealed that most DEGs in the roots and shoots were enriched in three main KEGG classes: metabolism, organismal systems and genetic information processing pathways. Among them, the number of DEGs was largest in metabolism category, including 4044 genes involved in different pathways such as biosynthesis of secondary metabolites, amino acid metabolism, carbohydrate metabolism, metabolism of cofactors and vitamins.

Abiotic stresses including salt stress usually result in high accumulation of sugars such as glucose, sucrose and galactose which has a great role in osmoregulation, homeostasis, stabilization of protein structure and carbon storage (Singh et al., 2015; Sami et al., 2016). Darko et al. (2019) investigated the content of different metabolites in wheat seedlings and found that plants exposed to NaCl exhibited higher levels of sucrose and galactose in root and shoot tissues, suggesting the participation of L-galactose in ascorbic acid pathway. Ascorbic acid has been shown to protect plants from stress-induced oxidative damage, acting as an antioxidant, and to enhance the growth and development of plants (Zhang et al., 2015). The results obtained in this study confirm these findings where two genes involved in galactose metabolism, and which encode beta-fructofuranosidase (INV) and raffinose synthase were identified to be regulated in the roots and shoots of Najran wheat under NaCl stress.

## 5 Conclusions

This investigation used RNA sequencing to profile salt-induced changes in the transcriptome of roots and shoots of a salt-tolerant wheat, the Najran Cultivar from Saudi Arabia. Previous studies used the same approach to study changes in the transcriptome of roots or shoots of different wheat plants under salt-stress or water-stress, to our knowledge no previous study analyzed these changes simultaneously in roots and shoots of same wheat plants. Our results, show that roots respond to a higher extent than shoots to salt-stress and that salt-stress induces organ specific responses as well as responses that are common to roots and shoots. These results suggest that an efficient attempt to improve tolerance to salt-stress should consider starting with optimizing antioxidants responses including the production of glutathione and phenolics particularly phenylpropanoids as well as galactose metabolism in roots and shoots. A transcriptomic approach such as the one used in this work is vital to map the global changes induced by salt-stress to the cellular functions and metabolic pathways. This needs however to be complemented with detailed expression- and functional analysis of key genes of these functions and pathways to inform an integrated and efficient approach to improve salt-tolerance in wheat and potentially other plant species.

## Data availability statement

The datasets presented in this study can be found in online repositories. The names of the repository/repositories and accession number(s) can be found in the article/**Supplementary Material**.

## Author contributions

These two authors contributed equally to this work and share first authorship, the inception of the project was made by TT, the two authors contributed to the design and implementation of the experiments as well as the analysis and interpretation of the results. NA wrote the first draft of the manuscript which was revised into its final version by TT. The two authors agree to be accountable for the content of the work. All authors contributed to the article and approved the submitted version.
