# Supplementary material for: Comparative transcriptomic profiling reveals differentially expressed genes and important related metabolic pathways in shoots and roots of a Saudi wheat cultivar (Najran) under salinity stress
Source: Front Plant Sci. 2023 Jul 28;14:1225541. doi: 10.3389/fpls.2023.1225541 (PMC10425591; doi:10.3389/fpls.2023.1225541)
Supplement: Supplementary file 1 [file DataSheet_1.docx]

Supplementary Material

**Comparative Transcriptomic Profiling Reveals Differentially Expressed Genes and Important Related Metabolic Pathways in a Saudi Wheat Cultivar (Najran) under Salinity Stress**

**Norah Alyahya^1,2^, Tahar Taybi^1^***

^1^School of Natural and Environmental Sciences, Newcastle University, Newcastle upon Tyne, NE17RU, UK

^2^Department of Biology, Faculty of Science, King Khalid University, Abha, Saudi Arabia

*** Correspondence:**Tahar Taybi
Tahar.taybi@newcastle.ac.uk

# Supplementary Figures and Tables

## Supplementary Figures

Figure S1: RNA-Seq data analysis workflow.


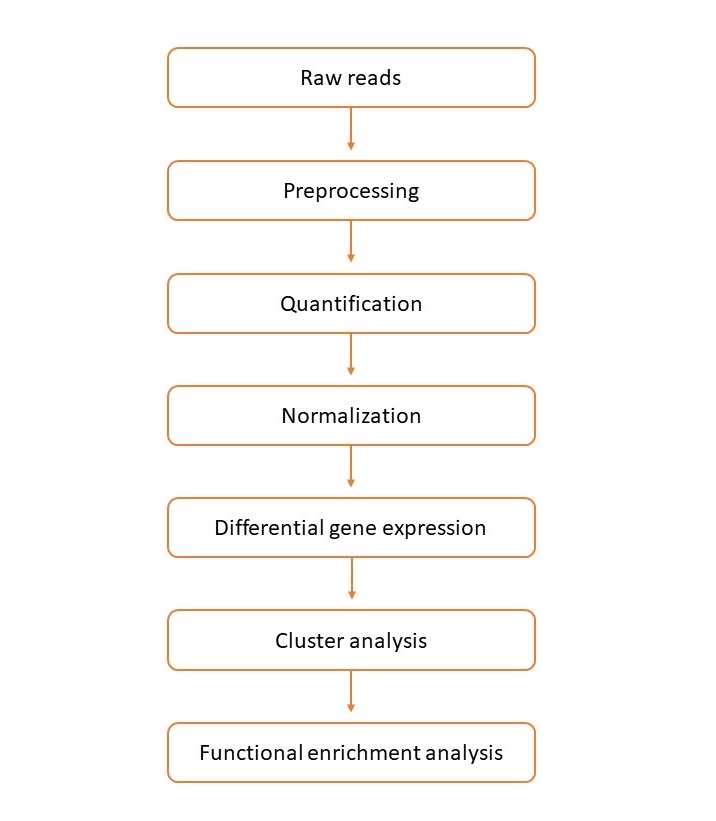


Figure S1. Workflow of RNA-seq data analysis in roots and shoots of Najran Wheat under salt-stress.

**
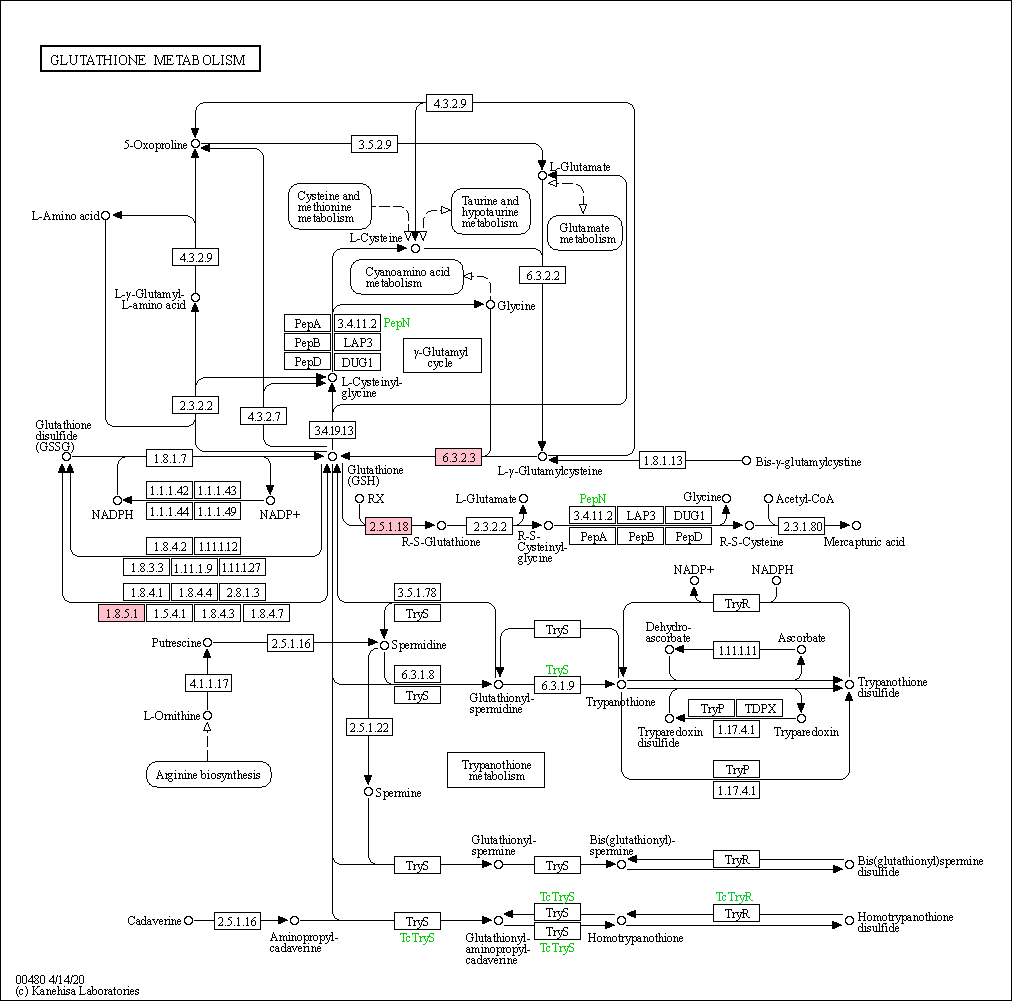
**

Figure S2: KEGG map of glutathione pathway regulated in the root of Najran wheat (*Triticum aestivum*) under salt stress conditions.

**
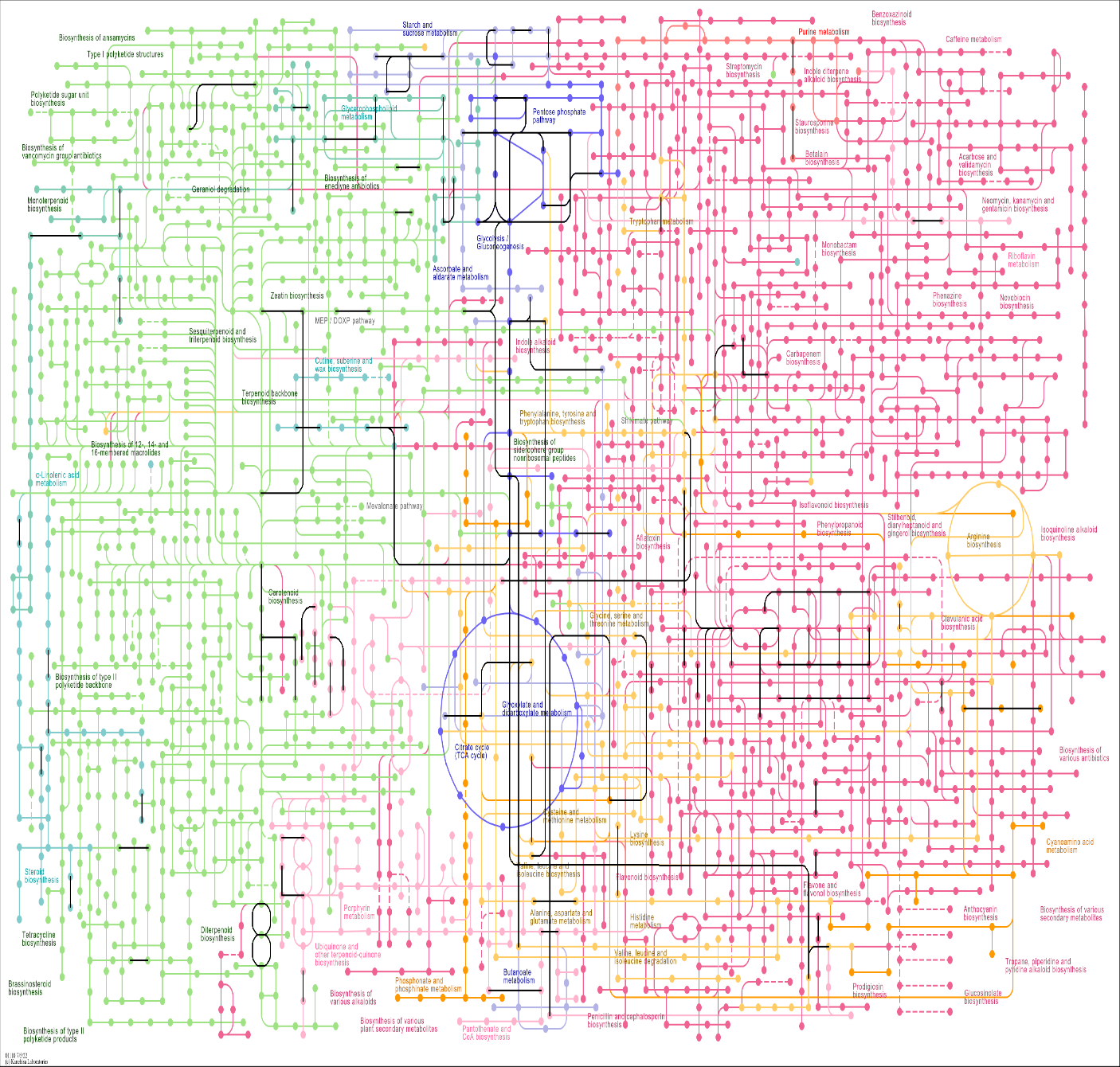
**

Figure S3: KEGG map of biosynthesis of secondary metabolites pathway regulated in the shoot of Najran wheat (*Triticum aestivum*) under salt stress conditions.

**
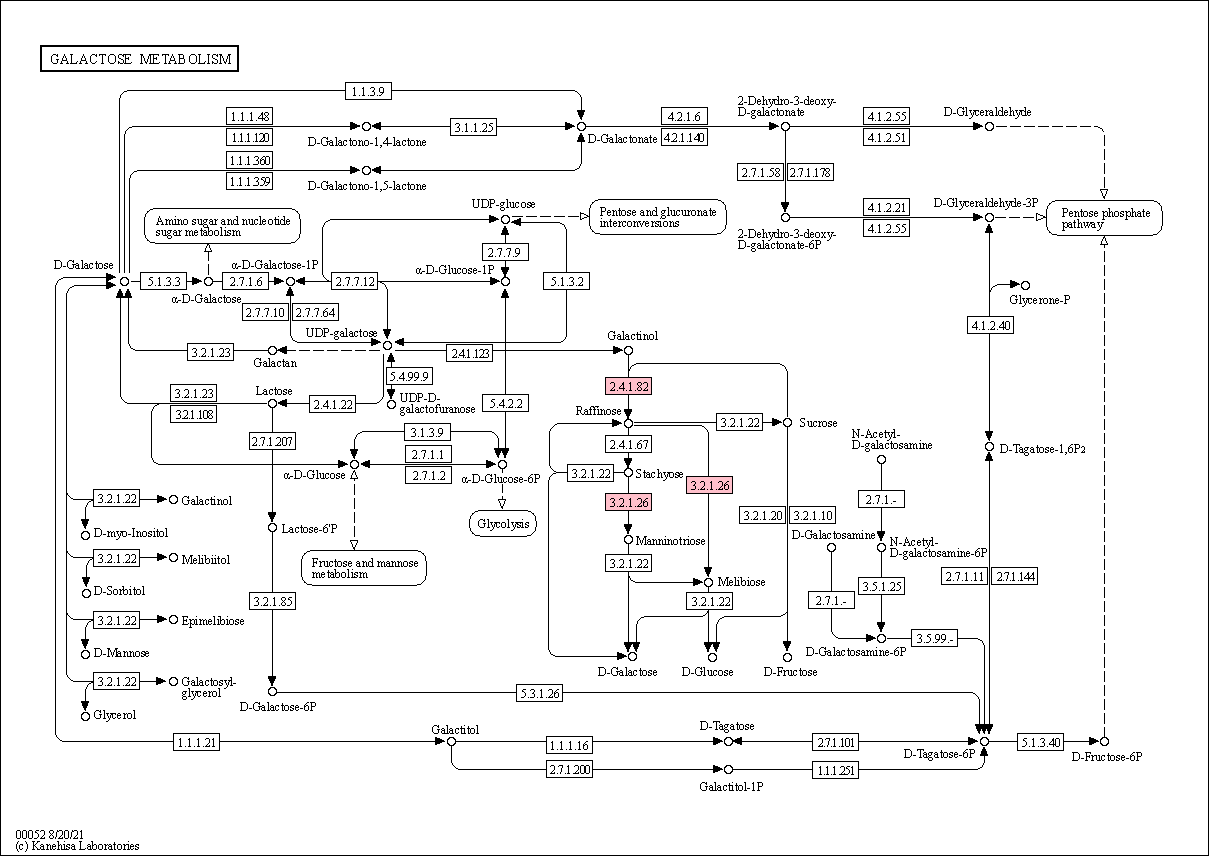
**

Figure S4: KEGG map of galactose pathway regulated in the root and shoot of Najran wheat (*Triticum aestivum*) under salt stress conditions.

## Supplementary Tables

Table S1. The eight DEGs randomly selected for the RT-qPCR

| **Gene ID** | **Gene name** | **Description** | **Log2 fold change (Root)** | **Log2 fold change (Shoot)** |
| --- | --- | --- | --- | --- |
| TraesCS2A02G033700 | Hsp90.1-A1 | Heat shock protein 90 [Source:UniProtKB/TrEMBL%3BAcc:F4Y589] | 5.07376836 | 7.365718292 |
| TraesCS4A02G431400 | *N/A* | Dirigent protein [Source:UniProtKB/TrEMBL%3BAcc:A0A1D5WYI3] | 4.987534773 | 7.024568308 |
| TraesCS3B02G395900 | TRAES_3BF065800020CFD_c1 | Delta-1-pyrroline-5-carboxylate synthase [Source:UniProtKB/TrEMBL%3BAcc:A0A077RXE4] | 1.542868089 | 4.129638563 |
| TraesCS5A02G348400 | *N/A* | Flavin-containing monooxygenase [Source:UniProtKB/TrEMBL%3BAcc:A0A1D5YP16] | 5.106458059 | 3.346230427 |
| TraesCS5B02G217500 | *N/A* | Glutamate receptor [Source:UniProtKB/TrEMBL%3BAcc:A0A1D5ZBQ4] | -1.6837149 | -2.820227478 |
| TraesCS2B02G333600 | *N/A* | Lipoxygenase [Source:UniProtKB/TrEMBL%3BAcc:A0A1D5U5U4] | -1.771863296 | -1.892795301 |
| TraesCS6A02G009100 | *N/A* | Bidirectional sugar transporter SWEET [Source:UniProtKB/TrEMBL%3BAcc:W5GC93] | 1.147319828 | -4.77364506 |
| TraesCS1B02G381500 | Wrab18 | ABA inducible protein [Source:UniProtKB/TrEMBL%3BAcc:Q7XAP5] | -1.189341477 | 5.395800134 |

Table S2. Oligonucleotide primers used in RT-qPCR confirmation.

| **Gene ID** | **Annotation** | **Primer Name** | **Primer Sequence (5’→3’)** |
| --- | --- | --- | --- |
| TraesCS2A02G033700  TraesCS4A02G431400  TraesCS3B02G395900  TraesCS5A02G348400  TraesCS5B02G217500  TraesCS2B02G333600  TraesCS6A02G009100  TraesCS1B02G381500  CJ705892  (Reference gene)  (Dudziak, 2020) | *TaHsp90.1-A1*  *TaDip*  *TaTRAES_3BF065800020CFD_c1*  *TaFCM*  *TaGLR*  *TaLIPX*  *TaBiSTS*  *TaWrab18*  *TaCJ70* | Hsp90.1-A1-**F**  Hsp90.1-A1-**R**  Dip**-F**  Dip-**R**  TRAES**-F**  TRAES**-R**  FCM**-F**  FCM**-R**  GLR-**F**  GLR-**R**  LIPX-**F**  LIPX-**R**  BiSTS**-F**  BiSTS**-R**  Wrab18**-F**  Wrab18-**R**  CJ70**-F**  CJ70**-R** | GCAGTGTCGTGTCTGTCCAT TCCCTCCGTTCTCACATCTC  TTCAGTATTCGACCGTGCTG  AGGCATAAACAACGGGACAC TCTCTCTGTGCGAGGAATGA  AGCATAGATGTACCGATGCAA  CAGAAACATTGCCATTCCAG  GGAGGACCGGTAACAAGAGT CGATGACGATGACGATGAAT  GCACCGACTGAGACTTCTGAC  GGGAATTTTTCAGCCTCGAT GATTCGGCCTTCGTAGTGAA GCAGTCATGCGTTCATGC AAAATAACTGGCTCGAGCATC  CATGCGTCCAATCTTGCTAA CATTACAGAACCGGACACGA  GCCTCAGTGGTAGGAGCATT  TTCAGCAAATGCGGTGGTTG |

Table S3. The significantly enriched pathways of identified DEGs in both root and shoot of *Triticum aestivum* cultivar (Najran) under control and salinity stress conditions.

|  | **Enriched KEGG Pathways** | **Main pathway** | **Pathway ID** | **Observed Significant Genes** | **Expected Significant Genes** |
| --- | --- | --- | --- | --- | --- |
| Salt treated root vs control root | Glutathione metabolism | Amino acid metabolism | path:taes00480 | 54 | 35 |
|  | Thiamine metabolism | Metabolism of cofactors and vitamins | path:taes00730 | 8 | 2 |
|  | Galactose metabolism | Carbohydrate metabolism | path:taes00052 | 28 | 14 |
| Salt treated Shoot vs Control Shoot | Phenylalanine metabolism | Amino acid metabolism | path:taes00360 | 18 | 8 |
|  | Phenylpropanoid biosynthesis | Biosynthesis of other  secondary metabolites | path:taes00940 | 26 | 12 |
|  | Biosynthesis of secondary metabolites |  | path:taes01110 | 130 | 96 |
|  | Starch and sucrose metabolism | Carbohydrate metabolism | path:taes00500 | 32 | 16 |
|  | Galactose metabolism |  | path:taes00052 | 24 | 9 |
|  | Protein processing in endoplasmic reticulum - | Genetic Information Processing; Folding, sorting and degradation | path:taes04141 | 43 | 20 |

Table S4. Most commonly identified KEGG pathways obtained from DEGs in roots and shoots of Najran Wheat under salt-stress.

| **Enriched KEGG Pathways** | **Pathway ID** | **Salt treated Root vs Control Root** | **Salt treated Shoot vs Control Shoot** |
| --- | --- | --- | --- |
| Glutathione metabolism | taes00480 | ✓ |  |
| Thiamine metabolism | taes00730 | ✓ |  |
| Galactose metabolism | taes00052 | ✓ | ✓ |
| Phenylalanine metabolism | taes00360 |  | ✓ |
| Phenylpropanoid biosynthesis | taes00940 |  | ✓ |
| Biosynthesis of secondary metabolites | taes01110 |  | ✓ |
| Starch and sucrose metabolism | taes00500 |  | ✓ |
| Protein processing in endoplasmic reticulum - | taes04141 |  | ✓ |
