# Supplementary material for: Comparative transcriptomic profiling reveals differentially expressed genes and important related metabolic pathways in shoots and roots of a Saudi wheat cultivar (Najran) under salinity stress
Source: Front Plant Sci. 2023 Jul 28;14:1225541. doi: 10.3389/fpls.2023.1225541 (PMC10425591; doi:10.3389/fpls.2023.1225541)
Supplement: Supplementary file 2 [file DataSheet_1.pdf]

Initial QC Report

Customer Name	Tahar Taybi
Institution/Company	Newcastle University , Biology School of Nature &Env Science
Project ID	21050-01-12-12v1

† contact custom-services with Project ID as reference for any questions

Contents

Sample ID Table & QC Results....3
TapeStation Image....4
Filename: 21050-01-IQC-TS-01-12-08052021.RNA....4
Sample QC Process & Guidelines....18
Sample QC Process....18
Sample QC Guidelines....18

Sample ID Table & QC Results

Sample #	Admera Health Sample ID	Customer Sample ID	Sample Type	Sample Volume (ul)	Admera Health Concentration (ng/ul)	Admera Health Total Quantity (ng)	RIN
1	21050R-01-01V1	1	RNA	20	550.00	11000	7.6
2	21050R-01-02V1	2	RNA	20	610.00	12200	8.1
3	21050R-01-03V1	3	RNA	20	478.00	9560	7.3
4	21050R-01-04V1	4	RNA	20	625.00	12500	6.5
5	21050R-01-05V1	5	RNA	20	347.50	6950	7.7
6	21050R-01-06V1	6	RNA	20	491.50	9830	6.8
7	21050R-01-07V1	7	RNA	20	905.00	18100	7.1
8	21050R-01-08V1	8	RNA	20	715.00	14300	7.9
9	21050R-01-09V1	9	RNA	20	840.00	16800	8.0
10	21050R-01-10V1	10	RNA	20	605.00	12100	6.8
11	21050R-01-11V1	11	RNA	20	277.00	5540	7.9
12	21050R-01-12V1	12	RNA	20	700.00	14000	7.8

TapeStation Image

Filename: 21050-01-IQC-TS-01-12-08052021.RNA

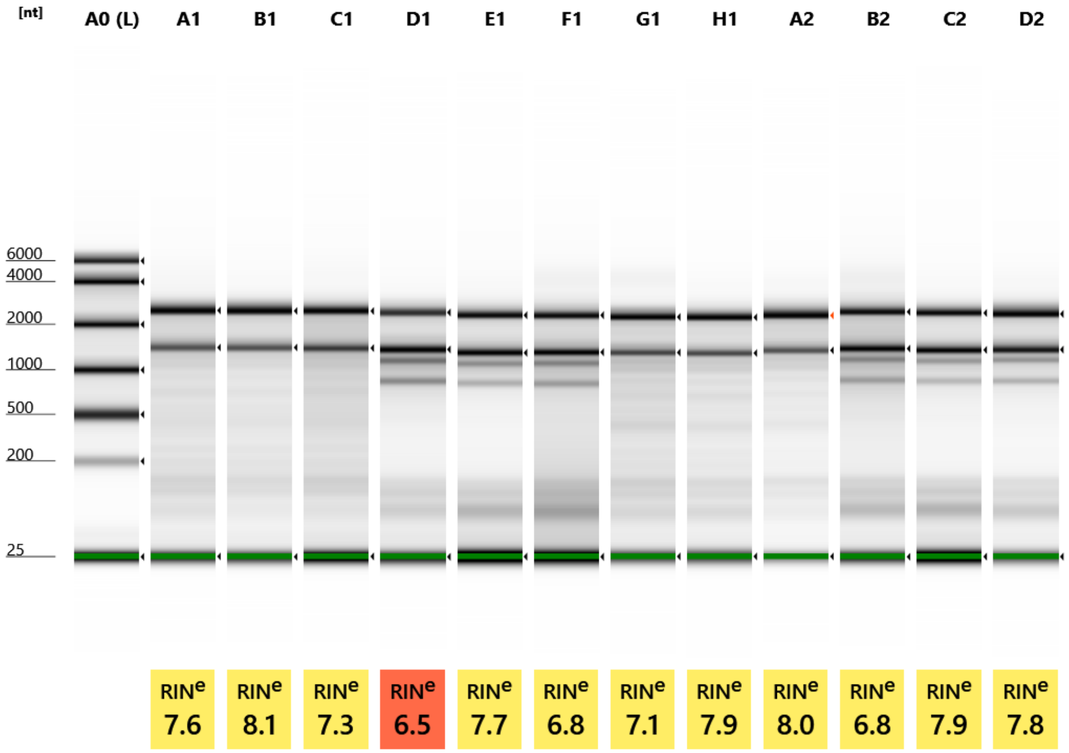

Default image (Contrast 50%), Image is Scaled to Sample

Sample Info

Well	RIN^e	28S/18S (Area)	Conc. [ng/µl]	Sample Description	Alert	Observations
A0	-	-	84.9	Electronic Ladder		Ladder
A1	7.6	1.3	87.6	21050R-01-01 V1		
B1	8.1	1.3	82.9	21050R-01-02 V1		
C1	7.3	1.1	69.7	21050R-01-03 V1		
D1	6.5	0.8	84.5	21050R-01-04 V1		
E1	7.7	1.0	49.1	21050R-01-05 V1		
F1	6.8	1.0	72.1	21050R-01-06 V1		
G1	7.1	1.2	98.9	21050R-01-07 V1		
H1	7.9	1.3	79.1	21050R-01-08 V1		
A2	8.0	1.4	128	21050R-01-09 V1		
B2	6.8	1.0	101	21050R-01-10 V1		
C2	7.9	1.0	50.8	21050R-01-11 V1		
D2	7.8	1.2	98.0	21050R-01-12 V1		

A0: Electronic Ladder

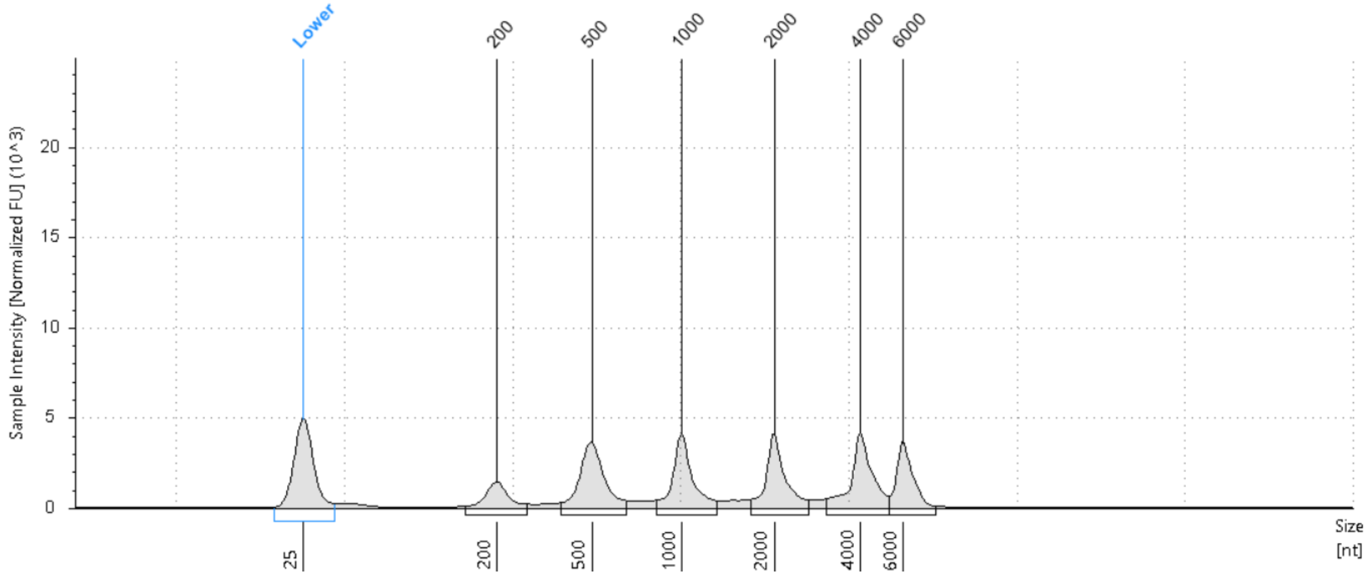

Sample Table

Well	RIN^e	28S/18S (Area)	Conc. [ng/µl]	Sample Description	Alert	Observations
A0	-	-	84.9	Electronic Ladder		Ladder

Peak Table

Size [nt]	Calibrated Conc. [ng/µl]	Assigned Conc. [ng/µl]	Peak Molarity [nmol/l]	% Integrated Area	Peak Comment	Observations
25	40.0	40.0	4710	-		Lower Marker
200	5.94	-	87.4	7.80		
500	15.9	-	93.6	20.88		
1000	14.2	-	41.8	18.62		
2000	13.8	-	20.3	18.11		
4000	15.5	-	11.4	20.38		
6000	10.8	-	5.31	14.22		

A1: 21050R-01-01 V1

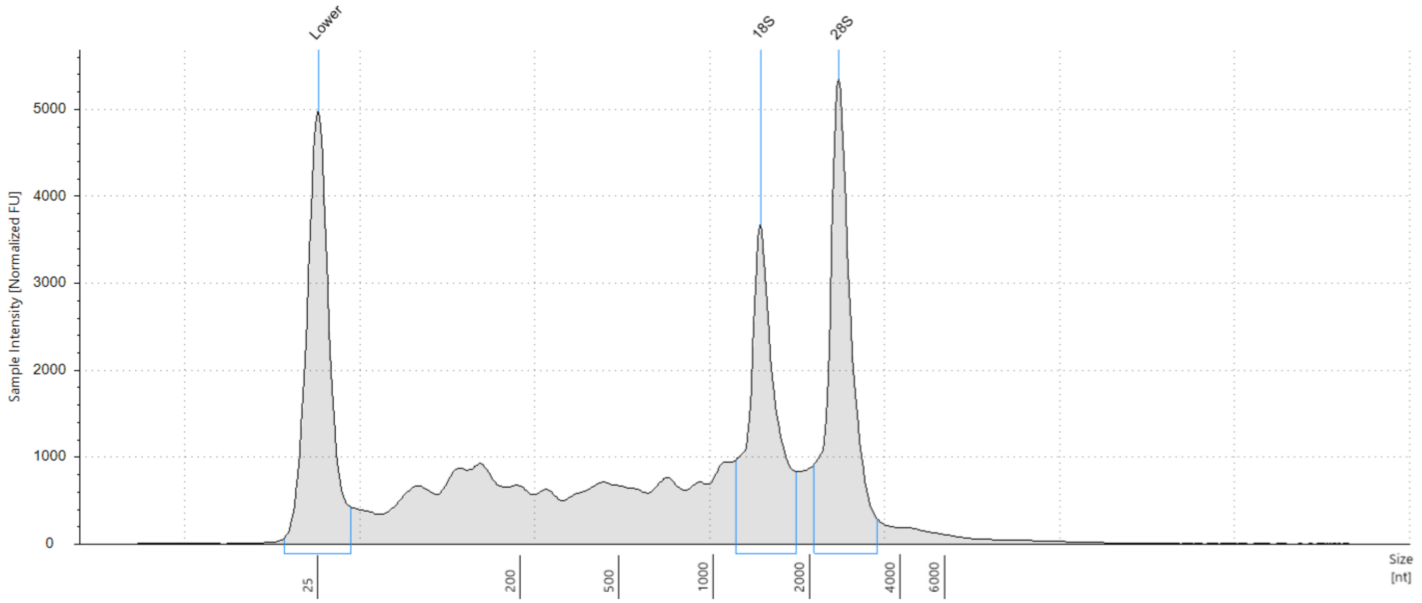

Sample Table

Well	RIN[e]	28S/18S (Area)	Conc. [ng/µl]	Sample Description	Alert	Observations
A1	7.6	1.3	87.6	21050R-01-01 V1		

Peak Table

Size [nt]	Calibrated Conc. [ng/µl]	Assigned Conc. [ng/µl]	Peak Molarity [nmol/l]	% Integrated Area	Peak Comment	Observations
25	40.0	40.0	4710	-		Lower Marker
1404	18.1	-	37.9	44.32		18S
2502	22.7	-	26.7	55.68		28S

B1: 21050R-01-02 V1

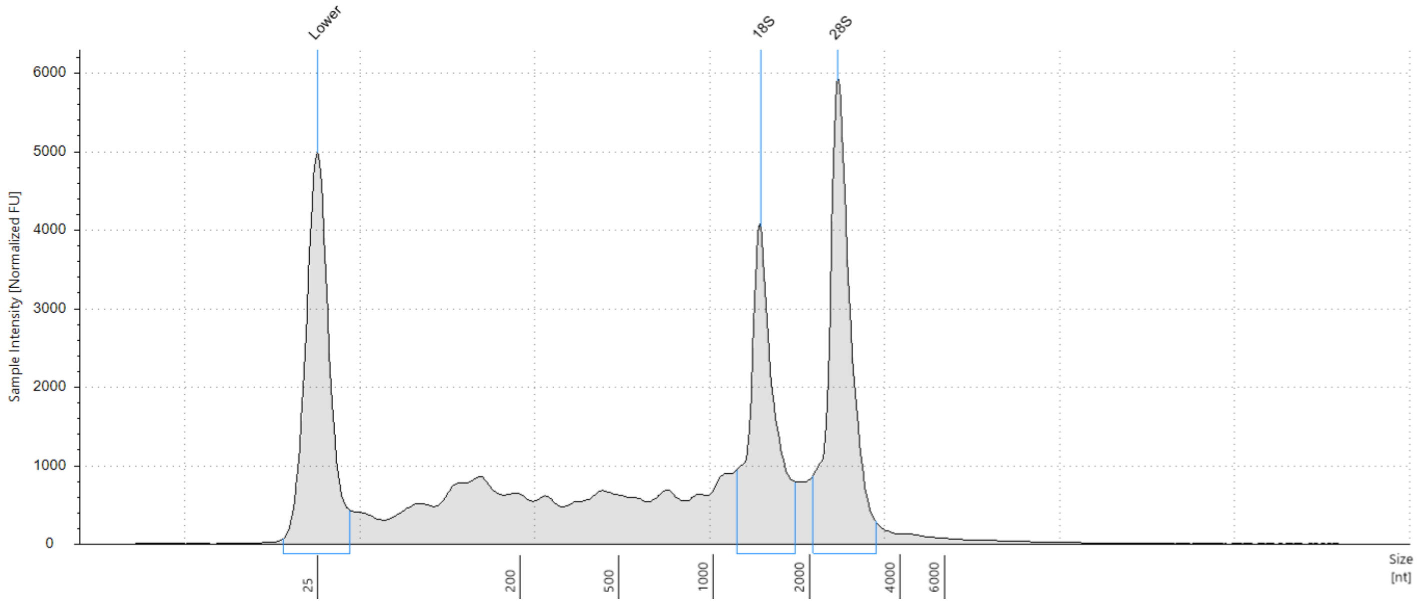

Sample Table

Well	RIN^e	28S/18S (Area)	Conc. [ng/µl]	Sample Description	Alert	Observations
B1	8.1	1.3	82.9	21050R-01-02 V1		

Peak Table

Size [nt]	Calibrated Conc. [ng/µl]	Assigned Conc. [ng/µl]	Peak Molarity [nmol/l]	% Integrated Area	Peak Comment	Observations
25	40.0	40.0	4710	-		Lower Marker
1408	17.8	-	37.2	42.68		18S
2489	23.9	-	28.2	57.32		28S

C1: 21050R-01-03 V1

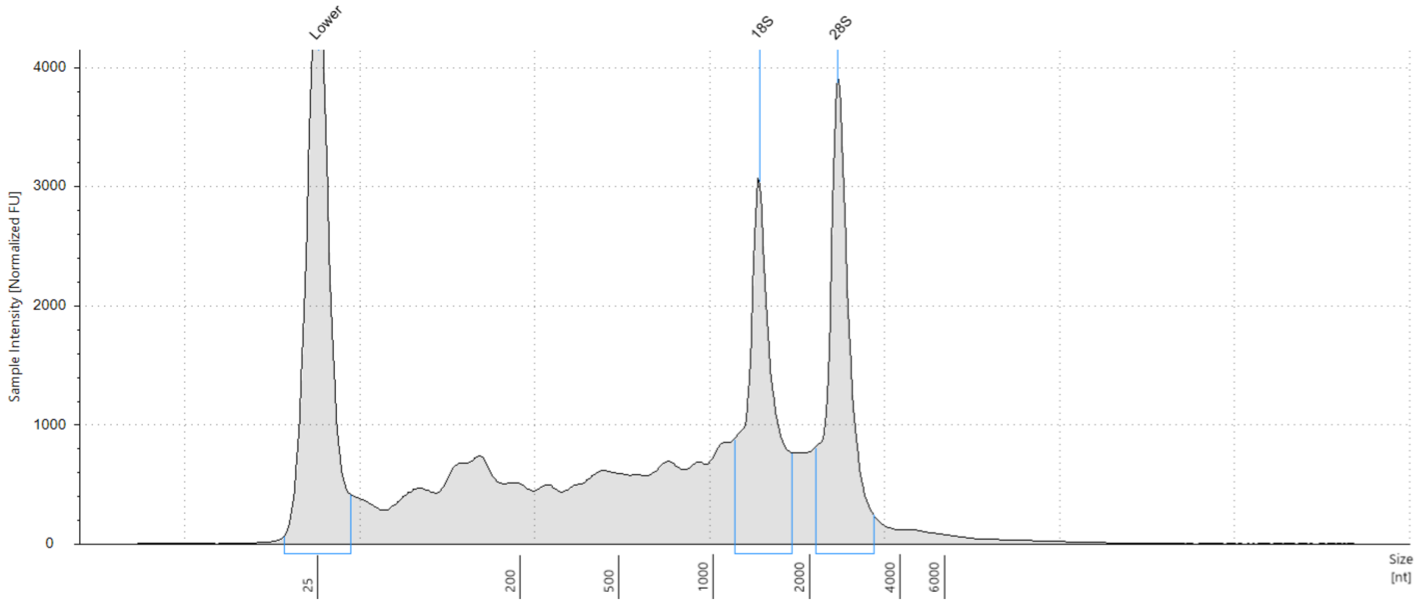

Sample Table

Well	RIN[e]	28S/18S (Area)	Conc. [ng/µl]	Sample Description	Alert	Observations
C1	7.3	1.1	69.7	21050R-01-03 V1		

Peak Table

Size [nt]	Calibrated Conc. [ng/µl]	Assigned Conc. [ng/µl]	Peak Molarity [nmol/l]	% Integrated Area	Peak Comment	Observations
25	40.0	40.0	4710	-		Lower Marker
1392	14.2	-	30.1	48.23		18S
2480	15.3	-	18.1	51.77		28S

D1: 21050R-01-04 V1

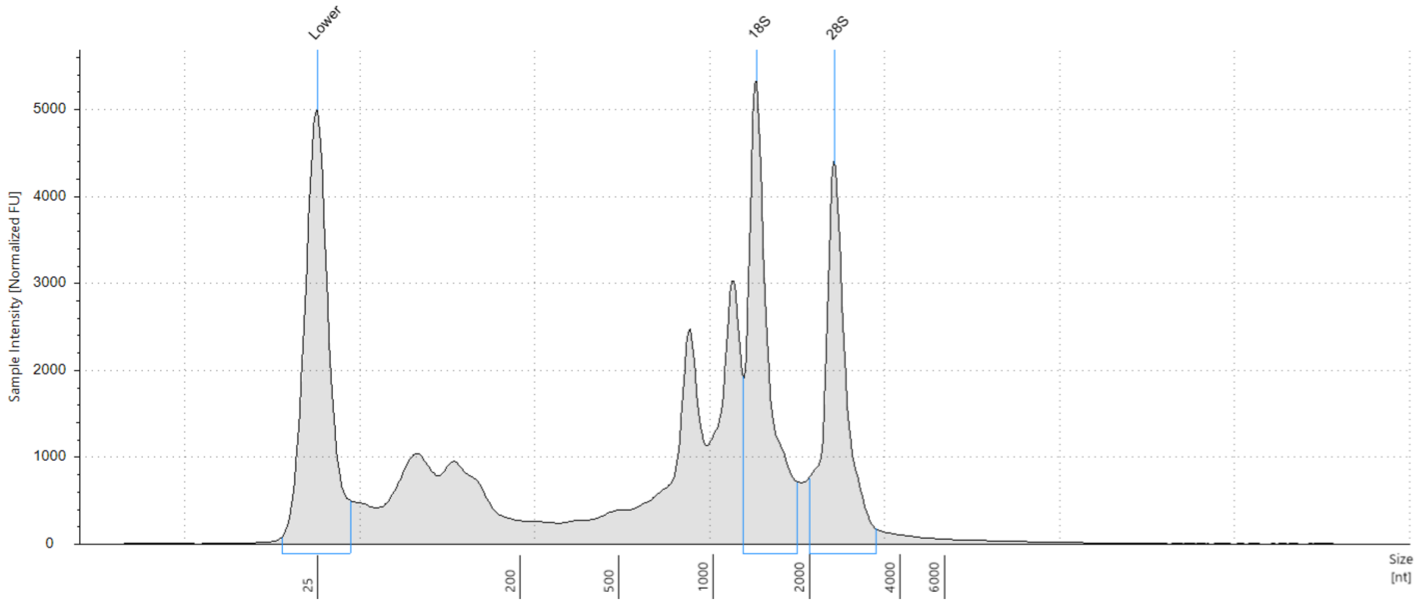

Sample Table

Well	RIN^{e}	28S/18S (Area)	Conc. [ng/µl]	Sample Description	Alert	Observations
D1	6.5	0.8	84.5	21050R-01-04 V1		

Peak Table

Size [nt]	Calibrated Conc. [ng/µl]	Assigned Conc. [ng/µl]	Peak Molarity [nmol/l]	% Integrated Area	Peak Comment	Observations
25	40.0	40.0	4710	-		Lower Marker
1364	19.8	-	42.6	54.37		18S
2420	16.6	-	20.2	45.63		28S

E1: 21050R-01-05 V1

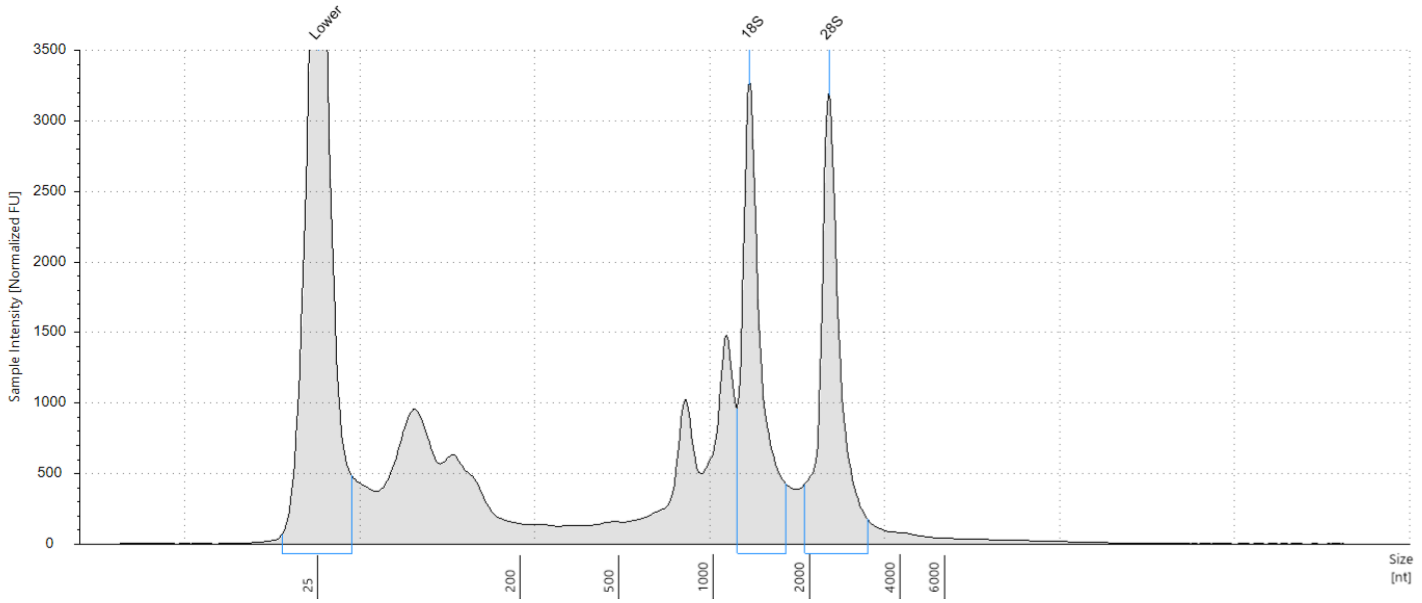

Sample Table

Well	RIN^e	28S/18S (Area)	Conc. [ng/µl]	Sample Description	Alert	Observations
E1	7.7	1.0	49.1	21050R-01-05 V1		

Peak Table

Size [nt]	Calibrated Conc. [ng/µl]	Assigned Conc. [ng/µl]	Peak Molarity [nmol/l]	% Integrated Area	Peak Comment	Observations
25	40.0	40.0	4710	-		Lower Marker
1296	10.8	-	24.5	50.13		18S
2317	10.8	-	13.7	49.87		28S edited

F1: 21050R-01-06 V1

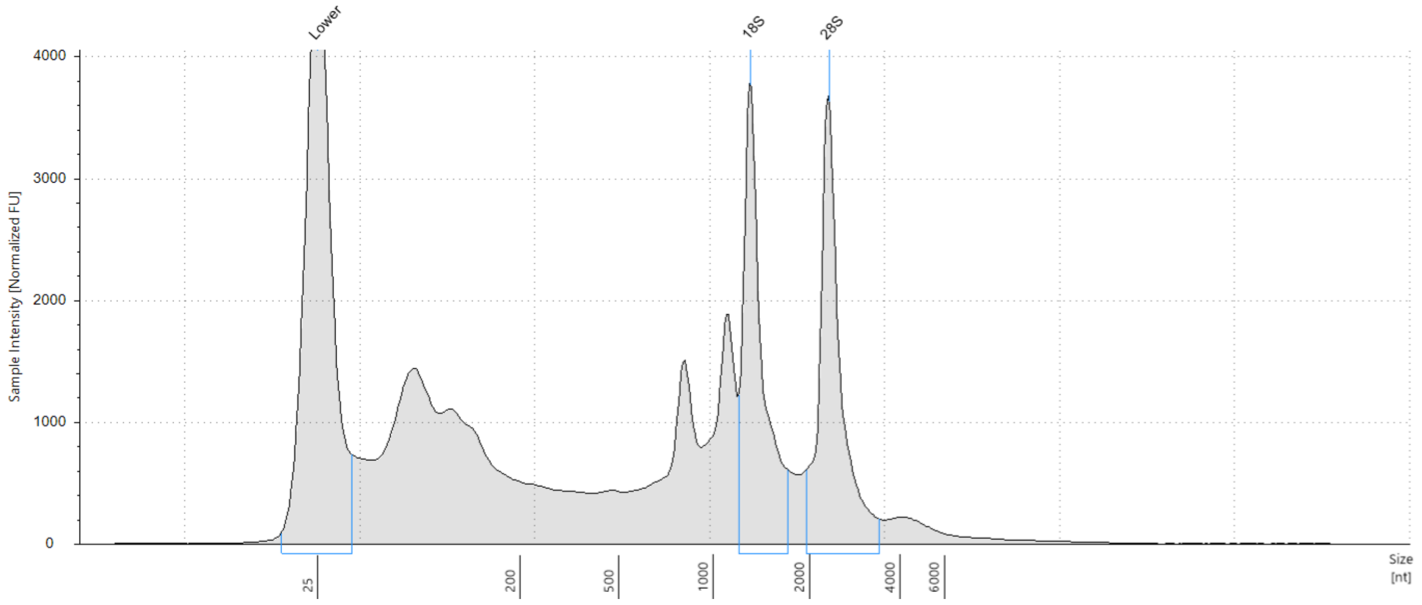

Sample Table

Well	RIN^e	28S/18S (Area)	Conc. [ng/µl]	Sample Description	Alert	Observations
F1	6.8	1.0	72.1	21050R-01-06 V1		

Peak Table

Size [nt]	Calibrated Conc. [ng/µl]	Assigned Conc. [ng/µl]	Peak Molarity [nmol/l]	% Integrated Area	Peak Comment	Observations
25	40.0	40.0	4710	-		Lower Marker
1309	12.2	-	27.5	49.79		18S
2319	12.3	-	15.6	50.21		28S edited

G1: 21050R-01-07 V1

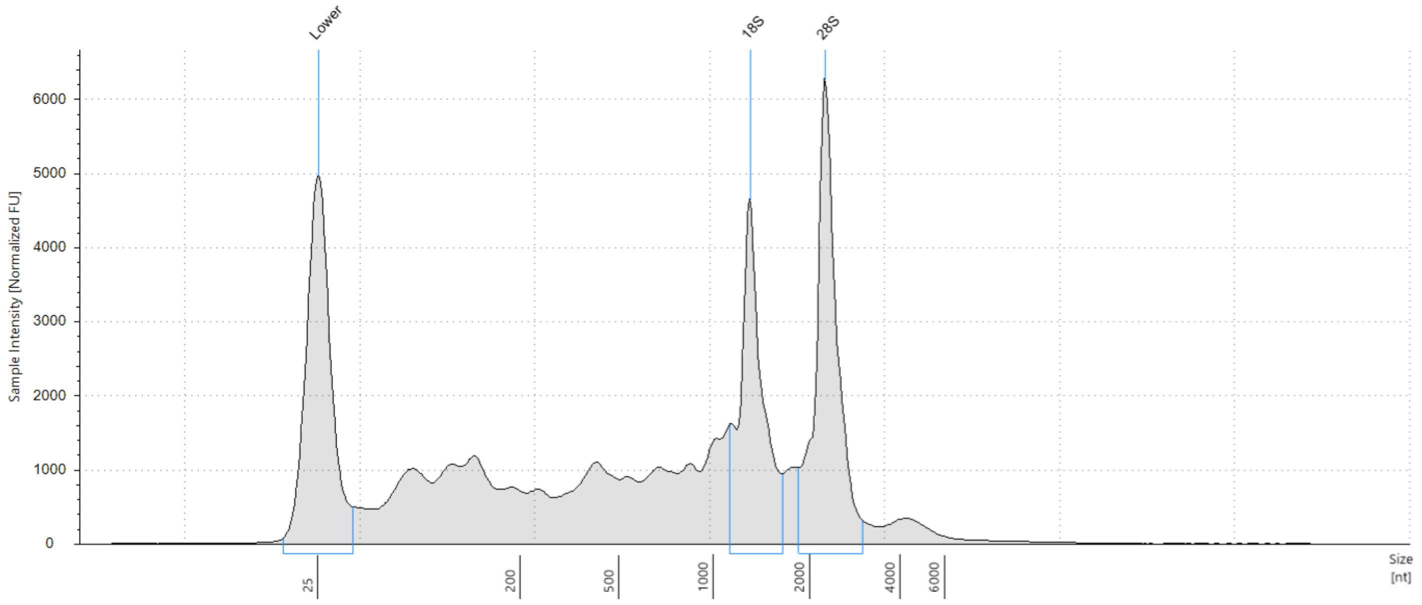

Sample Table

Well	RIN^{e}	28S/18S (Area)	Conc. [ng/µl]	Sample Description	Alert	Observations
G1	7.1	1.2	98.9	21050R-01-07 V1		

Peak Table

Size [nt]	Calibrated Conc. [ng/µl]	Assigned Conc. [ng/µl]	Peak Molarity [nmol/l]	% Integrated Area	Peak Comment	Observations
25	40.0	40.0	4710	-		Lower Marker
1298	18.6	-	42.1	44.65		18S edited
2244	23.0	-	30.2	55.35		28S edited

H1: 21050R-01-08 V1

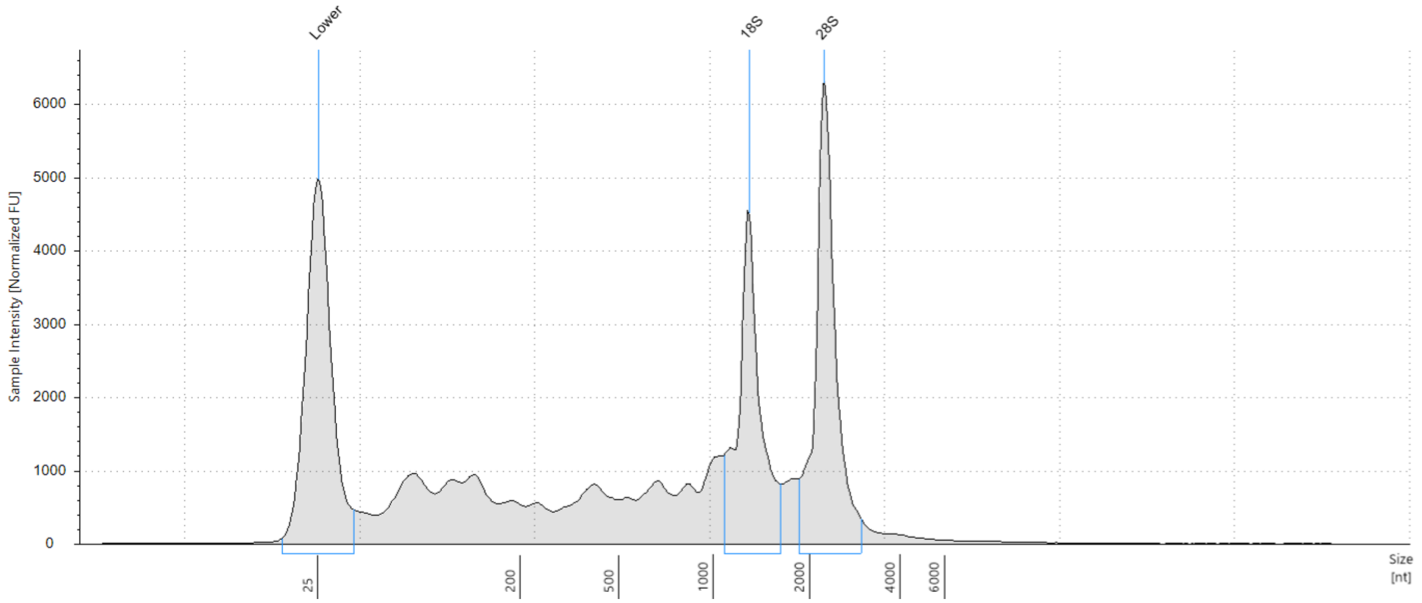

Sample Table

Well	RIN[e]	28S/18S (Area)	Conc. [ng/µl]	Sample Description	Alert	Observations
H1	7.9	1.3	79.1	21050R-01-08 V1		

Peak Table

Size [nt]	Calibrated Conc. [ng/µl]	Assigned Conc. [ng/µl]	Peak Molarity [nmol/l]	% Integrated Area	Peak Comment	Observations
25	40.0	40.0	4710	-		Lower Marker
1289	16.5	-	37.7	44.30		18S edited
2235	20.8	-	27.4	55.70		28S edited

A2: 21050R-01-09 V1

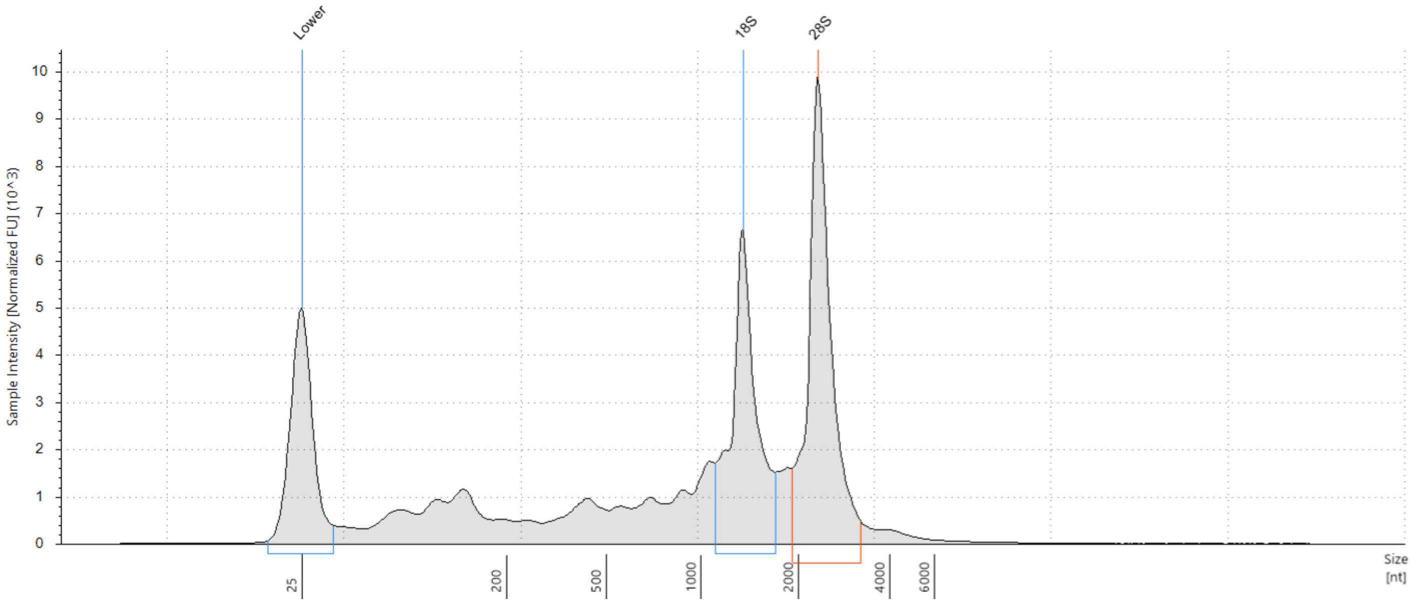

Sample Table

Well	RIN[e]	28S/18S (Area)	Conc. [ng/µl]	Sample Description	Alert	Observations
A2	8.0	1.4	128	21050R-01-09 V1		

Peak Table

Size [nt]	Calibrated Conc. [ng/µl]	Assigned Conc. [ng/µl]	Peak Molarity [nmol/l]	% Integrated Area	Peak Comment	Observations
25	40.0	40.0	4710	-		Lower Marker
1345	30.8	-	67.4	42.22		18S edited
2305	42.2	-	53.8	57.78		28S edited

B2: 21050R-01-10 V1

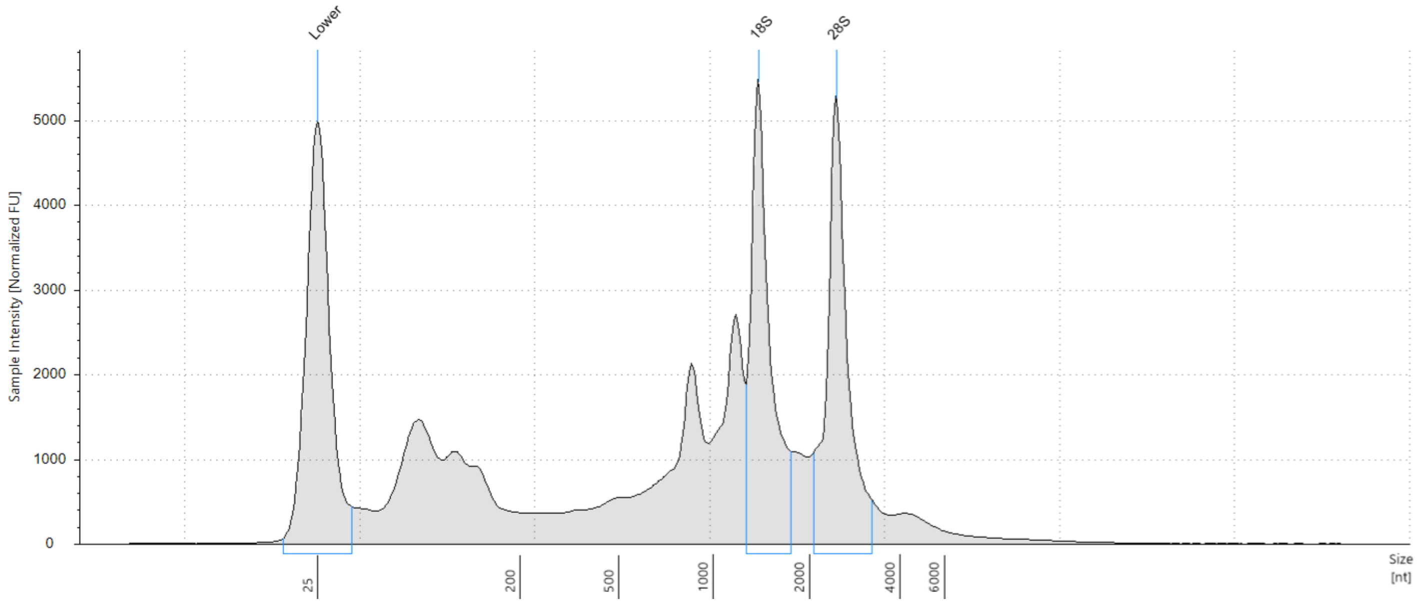

Sample Table

Well	RIN^{e}	28S/18S (Area)	Conc. [ng/µl]	Sample Description	Alert	Observations
B2	6.8	1.0	101	21050R-01-10 V1		

Peak Table

Size [nt]	Calibrated Conc. [ng/µl]	Assigned Conc. [ng/µl]	Peak Molarity [nmol/l]	% Integrated Area	Peak Comment	Observations
25	40.0	40.0	4710	-		Lower Marker
1382	19.5	-	41.4	49.56		18S
2448	19.8	-	23.8	50.44		28S

C2: 21050R-01-11 V1

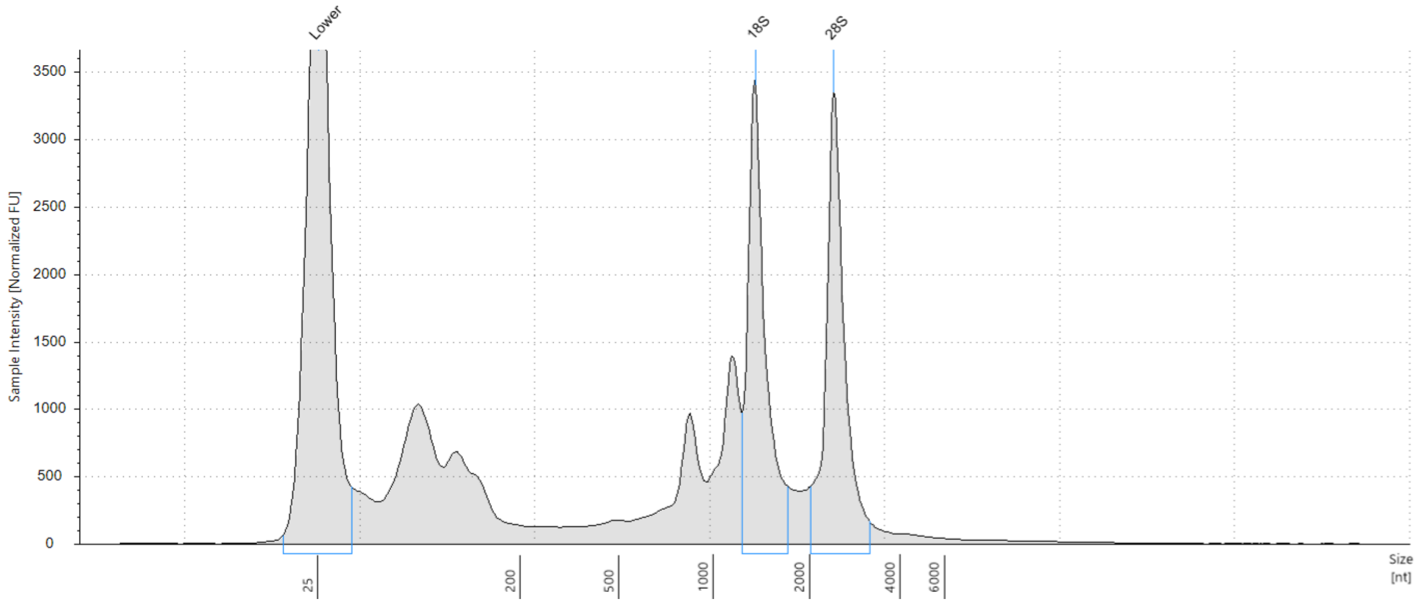

Sample Table

Well	RIN[e]	28S/18S (Area)	Conc. [ng/µl]	Sample Description	Alert	Observations
C2	7.9	1.0	50.8	21050R-01-11 V1		

Peak Table

Size [nt]	Calibrated Conc. [ng/µl]	Assigned Conc. [ng/µl]	Peak Molarity [nmol/l]	% Integrated Area	Peak Comment	Observations
25	40.0	40.0	4710	-		Lower Marker
1354	11.2	-	24.3	50.07		18S
2404	11.2	-	13.7	49.93		28S

D2: 21050R-01-12 V1

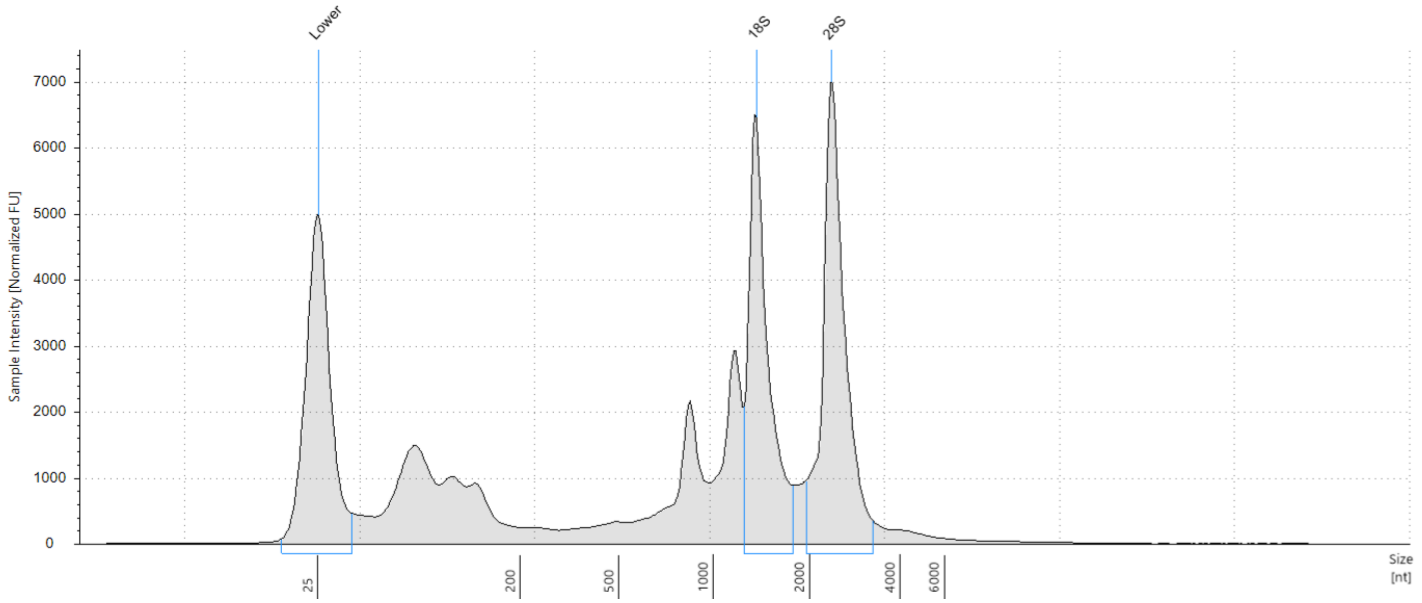

Sample Table

Well	RIN^{e}	28S/18S (Area)	Conc. [ng/µl]	Sample Description	Alert	Observations
D2	7.8	1.2	98.0	21050R-01-12 V1		

Peak Table

Size [nt]	Calibrated Conc. [ng/µl]	Assigned Conc. [ng/µl]	Peak Molarity [nmol/l]	% Integrated Area	Peak Comment	Observations
25	40.0	40.0	4710	-		Lower Marker
1357	22.7	-	49.1	45.51		18S
2357	27.1	-	33.9	54.49		28S

Sample QC Process & Guidelines

Sample QC Process

Admera Health qualifies samples based on the processes below:

DNA Quantity:

Qubit 2.0 DNA HS Assay (Life Technologies, Grand Island, NY)

DNA Quality

1% Standard agarose gel and/or Tapestation genomic DNA Assay (Agilent Technologies, CA, USA)

RNA Quantity:

Qubit RNA HS assay (ThermoFisher).

RNA Quality:

Bioanalyzer 2100 Eukaryote Total RNA Nano (Agilent Technologies, CA, USA)

Library Concentration & Quality:

Qubit 2.0 DNA HS Assay (ThermoFisher), QuantStudio ® 5 System (Applied Biosystems, USA)

Tapestation High Sensitivity D1000 Assay (Agilent Technologies, CA, USA)

Sample QC Guidelines

Service	Quantity Required*	Volume	Sample Type
16s_rRNA_Sequencing	≥50 ng	≥ 10 ul	gDNA
10x_Genomics	request for information	request for information	request for information
ATAC-seq	request for information	request for information	request for information
ChIPseq	50ng (ChIPed); 1 ug (Input)	≥ 20 ul	gDNA
ddRADseq	1 ug	≥ 20 ul	gDNA
HLA	1 ug	≥ 20 ul	gDNA
ITS1 ITS 2	≥50 ng	≥ 10 ul	gDNA
LiquidGx	request for information	request for information	request for information
Metagenomics	100ng	≥ 20 ul	gDNA
Pacbio	10ug	≥ 20 ul	High quality gDNA
RNAseq	1 ug	≥ 20 ul	RNA
Sequencing Only - Hiseq	4nM	≥ 10 ul	Library
Sequencing Only - Novaseq	3nM	≥ 30 ul	Library
Sequencing Only - Miseq	4nM	≥ 10 ul	Library
Sequencing Only - Nextseq	4nM	≥ 10 ul	Library
Sequencing Only - Custom Primers	0.3 uM	R1: 5ml; Ind: 4ml; R2: 2ml	
smRNAseq	1 ug	≥ 20 ul	RNA
qPCR	request for information	request for information	request for information
WES	1 ug	≥ 20 ul	gDNA
WGBS	1 ug	≥ 20 ul	gDNA
WGS	500ng-1ug	≥ 20 ul	gDNA
Amplicon_based	500 ng	≥ 10 ul	gDNA

*if cannot reach, please contact custom-services@admerehealth.com with your Project ID as reference
